# Orbital hybridization engineering, a built-in electric field, and simultaneous carbon layer charge rearrangement boost carrier migration for a superior cathode material

**DOI:** 10.1039/d6sc05330k

**Published:** 2026-08-11

**Authors:** Yanru Huo, Shuli Li, Mengxian Zhang, Xu Yang, Siyuan Li, Yu Feng, Hongen Shi, Shanghong Duan, Yanjun Chen

**Affiliations:** a School of Materials Science and Engineering, North University of China Taiyuan 030051 China yjchen@nuc.edu.cn sduan@nuc.edu.cn; b School of Energy and Power Engineering, Shanxi Key Laboratory of Efficient Hydrogen Storage & Production Technology and Application, North University of China Taiyuan 030051 China

## Abstract

To address the sluggish carrier kinetics of Na_3_V_2_(PO_4_)_3_ (NVP), we developed a multi-pronged synergistic modification strategy integrating “V3d–Pr4f” orbital hybridization to optimize the electronic structure, oxygen-vacancy-enriched heterogeneous NVP/NVPO to generate a built-in electric field, and an N/Br-codoped carbon layer that enables charge rearrangement. Notably, we introduce “V3d–Pr4f” orbital hybridization engineering to reconstruct the V3d electronic state, thus inducing modification of the “V–O” bond to accelerate carrier migration. EXAFS and simulation demonstrate the shortened bond length of V–O; ICOHP reveals that substituted Pr–O possesses stronger covalent bond energy than the primitive V–O bond, indicating a more stable Pr-VO_6_ framework. Meanwhile, ^23^Na-NMR and EPR together confirm the successful formation of an NVP/NVPO heterojunction with abundant oxygen vacancies, suggesting that lattice reconstruction has been achieved. Moreover, the difference in work function between NVP and NVPO promotes the spontaneous built-in electric field established at the interface, which has been comprehensively investigated by multiple spectroscopic and DFT calculations. Furthermore, charge rearrangement engineering is introduced into the coated carbon skeleton. ELF shows that charges accumulate at the N/Br sites after N/Br occupies C sites. The enriched charge is relatively attractive for O and induces O vacancies inside NVP. The optimized NVP/C@Pr-4% delivers a high capacity retention of 99.31% after 3000 cycles at 30C.

## Abstract 2

Herein, we developed a synergistic modification strategy to optimize Na_3_V_2_(PO_4_)_3_ including “V3d–Pr4f” orbital hybridization, NVP/NVPO heterojunction, and N/Br-codoped carbon layer coating.

## Graphical abstract

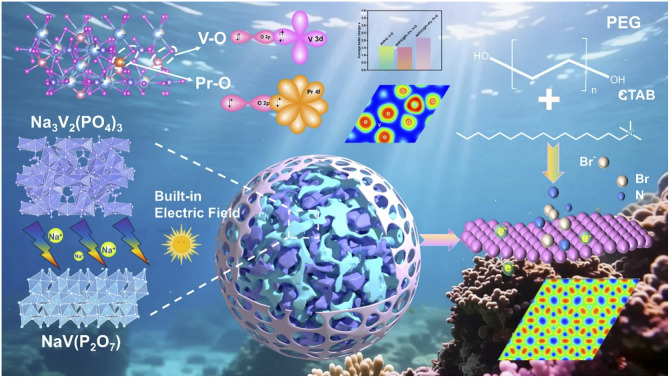

## 1. Introduction

Amid the global energy crisis and the urgent demand for sustainable energy storage, sodium-ion batteries (SIBs) are regarded as one of the most promising materials for large-scale energy storage systems, owing to the abundance of sodium resources,^1–7^ their relatively low cost, and their working mechanism, which is similar to that of lithium-ion batteries. Among various cathode materials, polyanionic compounds, particularly sodium vanadium phosphate (Na_3_V_2_(PO_4_)_3_, NVP), have attracted widespread attention from researchers due to their open three-dimensional ion diffusion channels, excellent cycling stability, and high discharge platform.^8^ However, polyanionic materials inherently suffer from low electronic conductivity, which severely limits their practical application in terms of high-rate performance and long-term cycling stability. In addition, single-phase NVP is prone to structural collapse during prolonged cycling, leading to capacity degradation.^9–12^

To overcome the aforementioned bottlenecks, researchers typically employ carbon-coating strategies to construct conductive networks or utilize cationic doping to optimize ion transport pathways. However, such single-modification approaches often struggle to simultaneously enhance charge transfer efficiency while suppressing interfacial side reactions. In recent years, the strategy of constructing heterostructures to form built-in electric fields that drive directional charge carrier migration has gradually become a research hotspot. Theoretically, doping with a rare earth element, owing to its unique 4f electron configuration and large ionic radius, is often employed to regulate lattice distortion and induce phase transitions. As a rare earth element, praseodymium (Pr) provides a wide channel for sodium ion transport through lattice distortion when doped into the NVP lattice and induces local changes in the chemical environment during the synthesis process, prompting the partial conversion of NVP to NVPO.^13^ This results in the formation of NVP/NVPO heterojunctions and an intrinsic electric field within the bulk material. In addition to regulating the bulk structure, the charge transport efficiency at the electrode–electrolyte interface is also crucial. Traditional carbon layers often suffer from issues such as a scarcity of defects, weak bonding, and poor ionic permeability. Atomic doping is an effective method to optimize the properties of carbon layers: nitrogen (N) doping can introduce a wealth of defect sites, enhancing the electronic conductivity of the carbon layer,^14,15^ whereas bromine (Br), as a halogen element, has a larger atomic radius that helps to increase the spacing between carbon layers,^16^ improving electrolyte wetting and accelerating sodium ion permeation; simultaneously, the high electronegativity of bromine enables it to modulate the charge distribution on the carbon layer surface.^17,18^ However, there is little research on the synergistic optimization of the carbon layer structure through codoping of bromine and nitrogen, combined with material-intrinsic heterojunctions. The key to further enhancing the performance of NVP materials lies in achieving phase transitions induced by bulk doping to create a synergistic effect between electric-field-driven and surface rapid ion channels.^19,20^

**Scheme 1:**
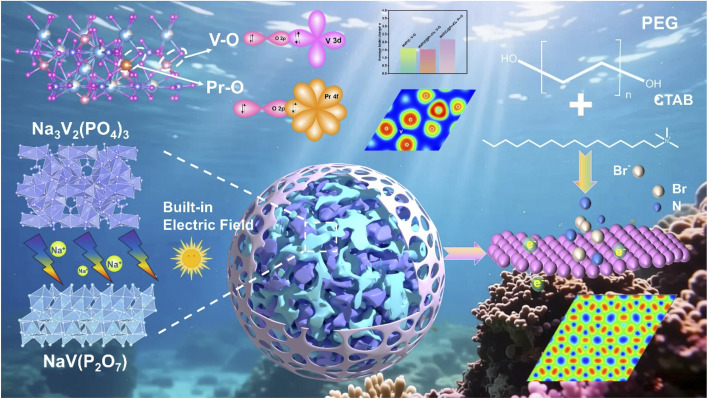
The modification strategy used in this work.

To this end, we have proposed an innovative multi-step synergistic modification strategy to prepare Pr-doped NVP/C. First, the introduction of Pr causes lattice expansion, which widens the framework structure and broadens the sodium ion migration channels, reducing the ionic diffusion barrier and enhancing ionic conductivity. Second, rare earth elements can induce a phase transformation in the material, causing some of the NVP to convert into NVPO, forming an NVP/NVPO heterojunction within the grains, which effectively drives the directional movement of sodium ions. Finally, we also introduce bromine/nitrogen (Br/N) codoping into the carbon layer on the material surface. Br incorporation increased the spacing between carbon layers and reduced the ionic diffusion barrier, and, together with N doping, facilitated the formation of a highly conductive and stable protective layer. This, in turn, suppressed interfacial side reactions and enhanced interfacial charge transport. This study not only reveals that Pr-doping stabilizes the crystal structure and that the built-in electric field of NVP/NVPO accelerates ion transport but also clarifies the core role of interface engineering in the bromine/nitrogen (Br/N)-codoped carbon layer, providing new insights and practical examples for the design of next-generation high-power, long-life cathode materials for sodium-ion batteries (Scheme 1).

## 2. Experiment

In this experiment, Br/N-codoped carbon layers and NVP cathodes modified by Pr bulk doping were synthesized using a hydrothermal method, with CTAB serving as the source of Br and N. Depending on the doping concentration, the resulting materials were designated NVP/C, NVP/C@Pr-1%, NVP/C@Pr-4% and NVP/C@Pr-7%. The detailed experimental procedure is provided in the SI.

## 3. Results and discussion

**Fig. 1:**
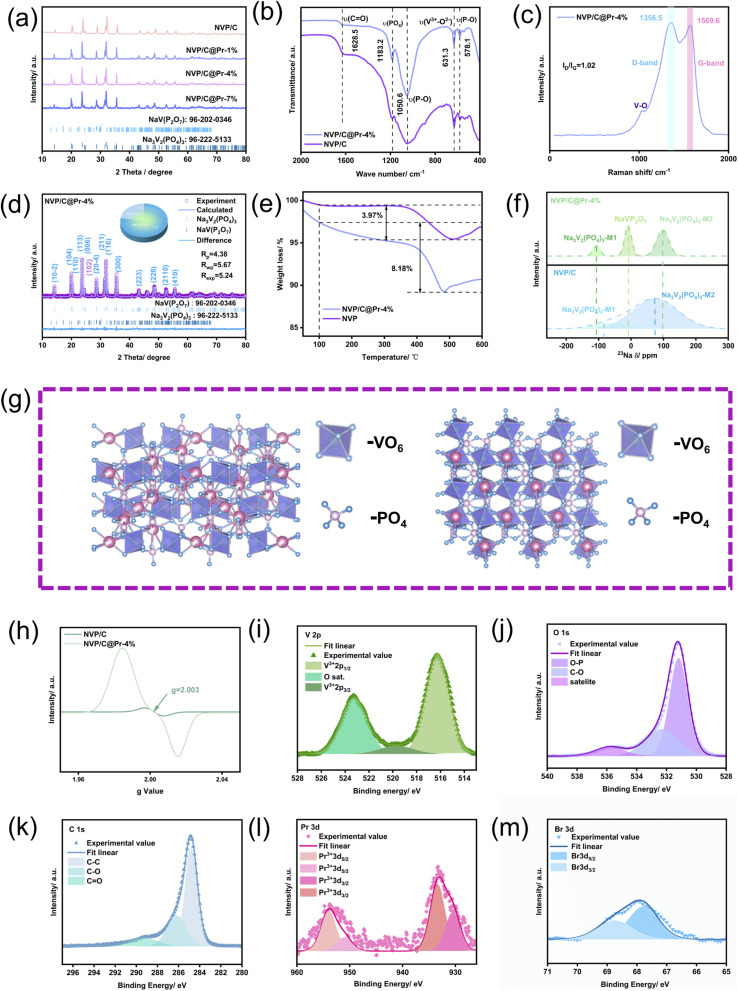
(a) X-ray diffraction (XRD) patterns of the NVP/C, NVP/C@Pr-1%, NVP/C@Pr-4% and NVP/C@Pr-7% samples. (b) FTIR of NVP/C@Pr-4% and NVP/C. (c) Raman curves of the NVP/C@Pr-4% sample. (d) XRD refinement of the NVP/C@Pr-4% sample. (e) TGA curves of the NVP/C@Pr-4% and NVP/C samples. (f) ^23^Na solid-state NMR spectra of the NVP/C@Pr-4% sample and the NVP sample. (g) Crystal structure models of the NVP and NVPO samples. (h) EPR curves of NVP/C@Pr-4% and NVP/C. High-resolution spectra of V 2p (i),O 1s (j), C 1s (k), Pr 3d (l) and Br 3d (m) of the NVP/C@Pr-4% sample.

A systematic analysis of the phase composition and crystal structure of the material was conducted using X-ray diffraction (XRD) (Fig. 1a). The diffraction patterns of NVP/C and samples with different Pr doping levels all exhibited sharp and intense characteristic peaks, indicating excellent crystallinity and the typical NASICON framework structure (belonging to the *R*3̄*c* space group). All diffraction peak positions were in strict agreement with the standard NVP card (PDF # 96-202-0346). Notably, in the NVP/C@Pr-4% sample, a weak diffraction peak was detected at 2*θ* = 27.02°. Upon calibration, this peak corresponds to the NaV(P_2_O_7_) (NVPO) phase (PDF # 96-22-5133), providing preliminary evidence that appropriate Pr doping induced the *in situ* formation of a secondary phase. To further verify the integrity of the microstructure, Fourier-transform infrared (FTIR) spectroscopy was utilized to compare the NVP/C@Pr-4% sample with the undoped sample (Fig. 1b). The absorption peaks at 528.1 cm^−1^ and 631.3 cm^−1^ were attributed to the bending vibration modes of P–O bonds in [PO_4_]^3−^ and V–O bonds in [VO_6_] octahedra, respectively. Meanwhile, the strong absorption bands at 1050.6 cm^−1^ and 1183.2 cm^−1^ corresponded to the symmetric and asymmetric stretching vibrations of P–O bonds. The clear presence of these key vibrational modes confirms that the introduction of Pr did not disrupt the structural stability of the phosphate polyhedral network. A detailed analysis of the structure of the carbon coating was carried out using Raman spectroscopy (Fig. 1c). In the NVP/C@Pr-4% sample, characteristic D (representing disordered carbon and defects) and G bands (representing graphitic sp^2^ carbon) were observed at around 1350 cm^−1^ and 1580 cm^−1^, respectively. The calculated *I*_D_/*I*_G_ value reached as much as 1.02. In carbon materials science, a value greater than 1 indicates that the carbon coating exists primarily in a highly disordered amorphous state, rich in edge defects. This “defect-rich” carbon network not only provides abundant short-range active pathways for Na^+^ intercalation and deintercalation at the interface but also significantly boosts the intrinsic electronic conductivity of the material by breaking symmetry. Combined with thermogravimetric analysis (TGA, Fig. 1e), the carbon coating content in NVP/C@Pr-4% was precisely determined to be 8.18 wt%, providing substantial support for the formation of an excellent long-range electron transmission network. The XRD data were further analyzed through full-pattern Rietveld refinement using HighScore software to quantify the phase composition of the system (Fig. 1d). The results revealed that NVP/C@Pr-4% consisted of 77% NVP as the main phase and 23% NVPO as the secondary phase, which corroborates the semi-quantitative results of XRD. Solid-state sodium nuclear magnetic resonance (^23^Na MAS NMR) spectroscopy was used to assess subtle changes in the local sodium environment (Fig. 1f). The undoped NVP/C sample exhibited two distinct resonance peaks corresponding to the two crystallographically inequivalent Na sites (M1 site Na1 and M2 site Na2) in the NASICON structure. In the NVP/C@Pr-4% sample, aside from retaining these two peaks, a new broadened resonance peak appeared in the high-field region, which precisely matched the chemical shift of the Na site in the NaV(P_2_O_7_) phase. This provides conclusive evidence for the presence of the NVPO secondary phase. Fig. 1g depicts the crystal structures of Na_3_V_2_(PO_4_)_3_ and NaV(P_2_O_7_): both consist of VO_6_ octahedra and PO_4_ tetrahedra connected *via* shared corners or edges to build robust three-dimensional framework networks. This high degree of lattice matching and structural similarity fundamentally lowers the interfacial energy between the two phases, providing a crystallographic basis for the atomic-scale coexistence of the two phases and the high-quality formation of heterointerfaces. Defect engineering is a key strategy for regulating ion transport dynamics. The electron paramagnetic resonance (EPR) spectrum (Fig. 1h) detected a strong unpaired electron signal at *g* = 2.003, characteristic of oxygen vacancies. The introduction of oxygen vacancies and other point defects acts as a “spatial expander” in the local lattice, effectively widening the three-dimensional diffusion pathways for Na^+^ and reducing the Na^+^ migration energy barrier through electrostatic interactions, thereby significantly enhancing ion diffusion kinetics. To comprehensively elucidate the chemical states and surface composition of the elements in the NVP/C@Pr-4% sample, high-resolution X-ray photoelectron spectroscopy (XPS) was performed.^21^ The XPS survey spectrum (Fig. S1) clearly identified characteristic peaks of core elements, such as Na, O, F, C, and P. In the Na 1s spectrum (Fig. S1a), a single symmetric peak was observed at 1071.04 eV, indicating that Na was in a uniform chemical environment. The V 2p spectrum (Fig. 1i) displayed main peaks at 516.23 eV and 523.25 eV, corresponding to the spin–orbit splitting of V^3+^ 2p_3/2_ and 2p_1/2_, respectively. Importantly, an additional peak was detected at 519.68 eV, further supporting the EPR results; this peak is attributed to perturbation of the local electronic state of neighboring V atoms due to the formation of lattice oxygen vacancies. The O 1s spectrum (Fig. 1j) was deconvoluted into three sub-peaks at 530.56 eV (lattice P–O bonds), 532.33 eV (surface defects or C=O bonds), and 534.94 eV (V–O bonds). Meanwhile, the C 1s spectrum (Fig. 1k) revealed the chemical complexity of the carbon coating, with peaks at 284.2 eV, 285.59 eV, and 288.57 eV corresponding to C–C/C=C, C–O, and C=O species, respectively. The presence of oxygen-containing functional groups contributes to improving the surface wettability of the material with the electrolyte. For the doped element, the Pr 3d spectrum (Fig. 1l) exhibited a complex multiplet structure resulting from spin–orbit coupling. By analyzing the characteristic peaks in the low-binding-energy region (3d_5/2_ at 930.4 eV/933.3 eV) and high-binding-energy region (3d_3/2_ at 950.8 eV/954 eV), it was confirmed that Pr existed stably in the +3 oxidation state in the lattice without any deviation in valence. Additionally, weak signals of Br 3d (3d_3/2_: 68.7 eV; 3d_5/2_ 67.7 eV, Fig. 1m) and N 1s (399.15 eV, Fig. S1b) were detected. These residual signals likely originated from trace amounts introduced during the precursor synthesis, but they do not affect the electrochemical activity or structure of the main material.^22^

**Fig. 2:**
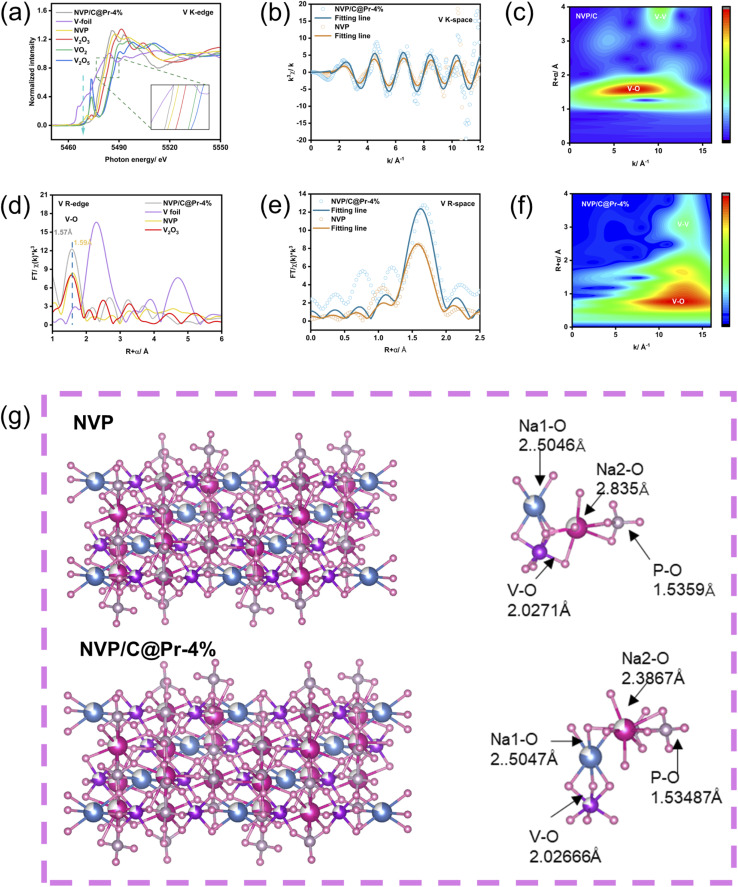
(a) K-edge XANES spectra of V. (b) Fitting data for *K*-space XANES spectra of V in the NVP/C@Pr-4% sample and NVP sample. (d) R-edge Fourier-transform EXAFS spectra of V. (e) Fitting data for *R*-space Fourier-transform EXAFS spectra of V in the NVP/C@Pr-4% sample and NVP sample. Wavelet transform of the *k*^3^-weighted EXAFS data of V in (c) the NVP/C sample and (f) the NVP/C@Pr-4% sample. (g) Ball-and-stick model of the NVP and NVP/C@Pr-4% samples.

The vanadium K-edge X-ray absorption near-edge structure (XANES) spectrum (Fig. 2a) reveals a clear shift in the absorption edge to lower energy for the NVP/C@Pr-4% sample compared to V_2_O_3_ and standard NVP.^21^ This indicates that the average valence state of vanadium in the sample is significantly lower than that in V_2_O_3_ or standard NVP. Moreover, the intensity and shape of the pre-edge feature have changed, suggesting reconstruction of the local coordination symmetry around vanadium atoms and a notable enhancement in the coordination environment. To quantitatively analyze this microstructural evolution, least-squares fitting was applied to extract amplitude information from the XANES spectrum (Fig. 2b). The data show that the amplitude of the NVP/C@Pr-4% sample significantly exceeds that of unmodified NVP. In XANES theory, an increase in amplitude typically reflects enhanced hybridization between the central atom and ligands as well as optimized coordination geometry. This confirms that the modified sample possesses a more compact and highly ordered local coordination environment. The increased amplitude is fundamentally attributed to the strengthened V–O coordination bonds and the improved local structural order, which profoundly demonstrates the positive effect of trace Pr doping in stabilizing and optimizing the vanadium oxygen octahedral framework. To further obtain real-space structural parameters, such as bond lengths and coordination numbers, extended X-ray absorption fine structure (EXAFS) analysis was conducted, and the radial distribution functions (*R*-space) are shown in Fig. 2d and e. In the *R*-space (not phase-shift corrected), the primary peak corresponding to the first coordination shell (V–O bond) for the NVP/C@Pr-4% sample shifted notably to lower *R* compared to unmodified NVP, with the average bond length shortening from 1.59 Å to 1.57 Å. This suggests a substantial contraction of the V–O chemical bond. Although the ionic radius of Pr^3+^ (0.101 nm) is larger than that of V^3+^ (0.064 nm), the observed bond shortening here does not result from lattice expansion due to geometric factors but rather from reconstruction of the local electronic structure. Furthermore, the intensity of the coordination peak at 1.62 Å for NVP/C@Pr-4% increased significantly. In EXAFS theory, increased peak intensity corresponds directly to a reduction in the Debye–Waller factor, which strongly indicates a decrease in local structural disorder and higher atomic ordering. This trait is crucial for suppressing structural degradation during long-term cycling and enhancing the intrinsic stability of the material. To overcome spatial interferences that arise in traditional Fourier transforms when distinguishing between atomic pairs with similar radial distances but different backscattering paths, a *k*^3^-weighted wavelet transform (WT) was introduced for two-dimensional analysis of the EXAFS data (Fig. 2c and f). WT maps coordinate *R*-space (radial distance) with *k*-space (photoelectron wave vector) to precisely decouple the contributions from different coordination shells. The analysis shows that the V–O coordination contour lines in the WT map for the NVP/C@Pr-4% sample exhibit clear stretching towards higher *k*-values. According to EXAFS principles, shifts to higher *k*-values are characteristic frequency-domain representations of bond length contraction, further corroborating the V–O bond shortening in reciprocal space. Simultaneously, the overall increase in signal intensity in the WT map further supports the optimization of the coordination environment and improvement in crystalline framework symmetry. Wavelet transform analysis eliminates phase ambiguities inherent in Fourier transforms and provides more comprehensive and multidimensional structural information for elucidating the local atomic configurations of the material. Fig. 2g illustrates the microscopic crystal structure models of NVP and NVP/C@Pr-4%, which were constructed based on the Rietveld refinement results of the XRD data (Fig. S2). The macroscopic unit cell parameters obtained from the refinements are in excellent agreement with the trends in local V–O bond length changes observed from the EXAFS spectroscopic analysis. This visually confirms the physical contraction of the V–O bond. Investigation into the underlying physical mechanism reveals that this short-range bond contraction arises from a synergistic interplay of multiple physical and chemical processes.^23^ On the one hand, lattice incorporation of the Pr element disrupts the original charge distribution, inducing electronic rearrangement within the lattice. The high polarizability of Pr leads to the redistribution and concentration of the electron cloud density around the V–O bond region, significantly enhancing the covalency of the V–O bond. This ultimately manifests as V–O bond contraction, providing a robust structural and mechanical foundation for the construction of a high-stability NASICON framework.

**Fig. 3:**
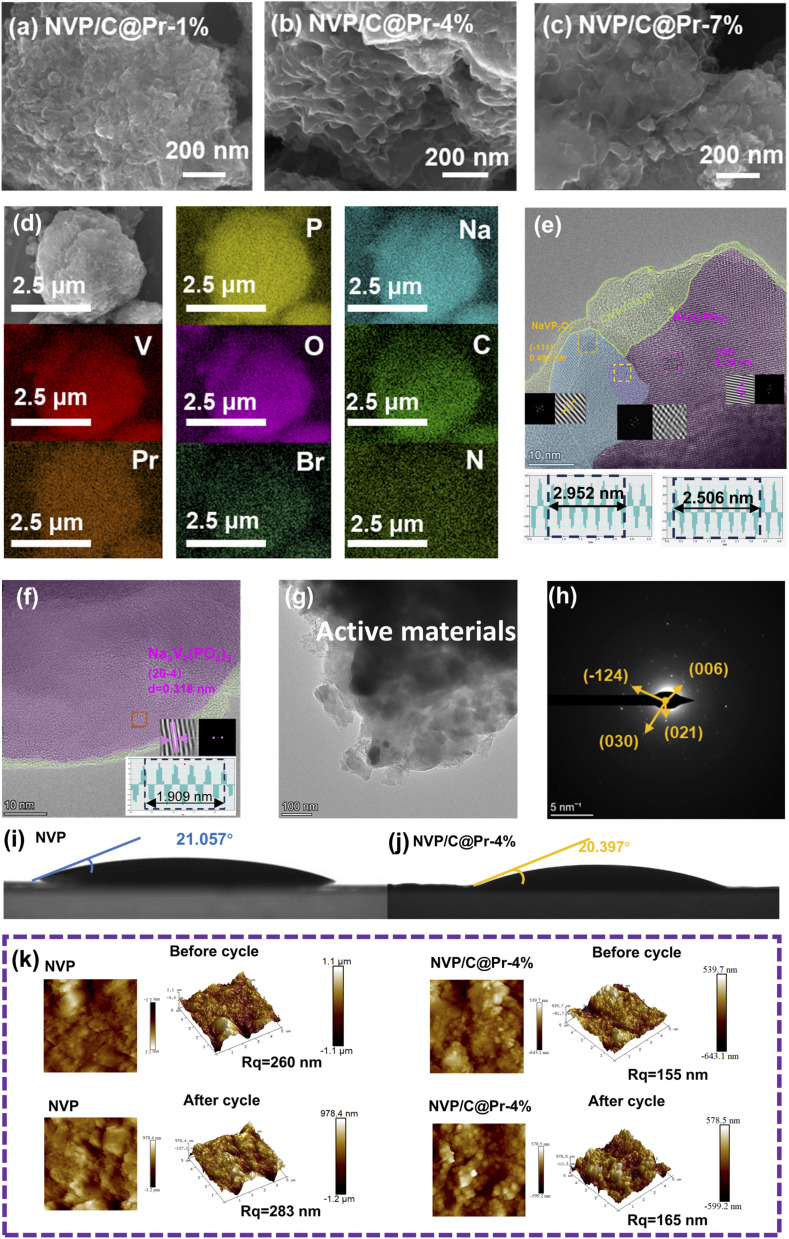
(a–c) SEM photographs of NVP/C@Pr-1%, NVP/C@Pr-4%, NVP/C@Pr-7% at 200 nm. (d) Mapping spectra of each element in NVP/C@Pr-4%. (e–g) TEM photographs of NVP/C@Pr-4% at different resolutions. (h) Selected electron diffraction patterns (i and j). Contact angle characterisation results for NVP/C and NVP/C@Pr-4%. (k) AFM images of NVP and NVP/C@Pr-4% before cycles and after 100 cycles.

The surface morphology of the NVP/C@Pr-1%, NVP/C@Pr-4%, and NVP/C@Pr-7% samples was systematically characterized using scanning electron microscopy (SEM) (Fig. 3a–c), directly revealing the influence of different Pr doping levels on the morphological evolution and structural regulation of the material. Observations show that the NVP/C@Pr-4% sample exhibits a rich micro–nano hierarchical porous structure. The formation of these pores can be attributed to a synergistic dual effect: first, local lattice distortions and micro-stress release are induced by the incorporation of larger Pr^3+^ ions into the lattice; second, porosity is created during high-temperature pyrolysis of the organic carbon precursor, caused by the escape of volatile gases. This distinctive porous morphology not only significantly increases the specific surface area of the material but also constructs a three-dimensional ion transport pathway that facilitates electrolyte infiltration and rapid deintercalation of Na^+^, thereby greatly enhancing the ionic transport kinetics of the material. In contrast, the NVP/C@Pr-1% sample, due to insufficient doping, displays a relatively dense and smooth surface without effective mass-transfer pores. For the NVP/C@Pr-7% sample, over-doping disrupts the original nucleation and growth dynamics, leading to severe particle aggregation. These comparisons clearly indicate that 4% is the optimal Pr doping ratio.

To further investigate the microscopic spatial distribution of the elements, SEM-EDS and scanning transmission electron microscopy coupled with energy-dispersive X-ray spectroscopy (STEM-EDS) analyses were performed (Fig. 3d). Elemental mapping clearly shows that key elements, such as P, Na, V, O, C, Pr, Br, and N, are uniformly distributed both within the particles and on the surface. This homogeneous distribution confirms that Pr^3+^ ions have been successfully and uniformly incorporated into the lattice sites of the sodium vanadium phosphate framework, rather than being simply physically attached to the surface. Furthermore, it confirms that the amorphous carbon layer uniformly and compactly encapsulates the outer material in a “three-dimensional wrapping” manner. A detailed quantitative analysis of the elemental composition can be found in SI Table S1. Transmission electron microscopy (TEM) was used to further reveal the fine structural characteristics of the material at the atomic scale (Fig. 3e–h). High-resolution transmission electron microscopy (HRTEM) images (Fig. 3e) reveal a low-contrast amorphous carbon coating layer, within which distinct lattice fringes with spacings of 0.492 nm and 0.358 nm were observed. Careful calibration attributes these fringes to the (111) plane of the NaVP_2_O_7_ phase and the (202) plane of the Na_3_V_2_(PO_4_)_3_ phase, respectively. The coexistence of these lattice fringes in real space directly confirms the formation of a heterostructure. Fig. 3f further magnifies the uniform carbon coating layer, which has a thickness of approximately 4 nm—an optimal scale for balancing electronic conductivity and ionic diffusion. Beneath the carbon layer, lattice fringes with a spacing of 0.318 nm can be observed, corresponding to the (20−4) plane of Na_3_V_2_(PO_4_)_3_. Fig. 3g shows the overall morphology of the particles at a lower magnification. The selected area electron diffraction (SAED) pattern (Fig. 3h) exhibits sharp, well-defined single-crystal diffraction spots with a highly periodic and symmetric arrangement. These spots, after calibration, correspond to the (−124), (006), (030), and (021) planes. The high degree of sharpness and the absence of diffuse scattering in the diffraction spots strongly demonstrate that the modified material possesses extremely high crystallinity and long-range order. This indicates that the Pr doping and carbon coating processes, while introducing defects and porosity, do not compromise the intrinsic crystal framework of the material. On the contrary, the local atomic arrangement is optimized, enhancing the overall structural integrity of the crystal to some extent. The wettability of the electrode surface by the electrolyte plays a direct role in determining the efficiency of interfacial charge transfer. Contact angle measurements (Fig. 3i and j) show that the contact angle of the NVP/C@Pr-4% electrode is significantly reduced. This improved wettability is attributed to the effect of the porous morphology, which reduces the charge transfer resistance (*R*_ct_) at the solid–liquid interface and accelerates reaction kinetics. To evaluate the structural stability of the material under electrochemical stress, *in situ* atomic force microscopy (AFM) was used to characterize the evolution in surface morphology of the NVP and NVP/C@Pr-4% electrodes before and after cycling (Fig. 3k). The 3D morphological data reveal that, after 100 cycles, the surface roughness (*R*_a_) of the NVP/C@Pr-4% electrode changes by only 10 nm, significantly lower than the 23 nm observed for the unmodified NVP electrode. This exceptional retention of surface morphology is attributed primarily to the synergistic effect of three mechanisms: the three-dimensional conductive network formed by the carbon coating layer acts as an “elastic buffer layer” during Na^+^ deintercalation, effectively mitigating volume stress and suppressing secondary particle pulverization and fracture. The rigid framework structure constructed by Pr^3+^ doping enhances the mechanical stability of the material at the bulk level, effectively restraining lattice distortions during cycling. The heterogeneous coexistence of the NVP and NVPO phases helps to distribute and release localized stresses at the interface, further endowing the material with exceptional structural stability during long-term cycling.

**Fig. 4:**
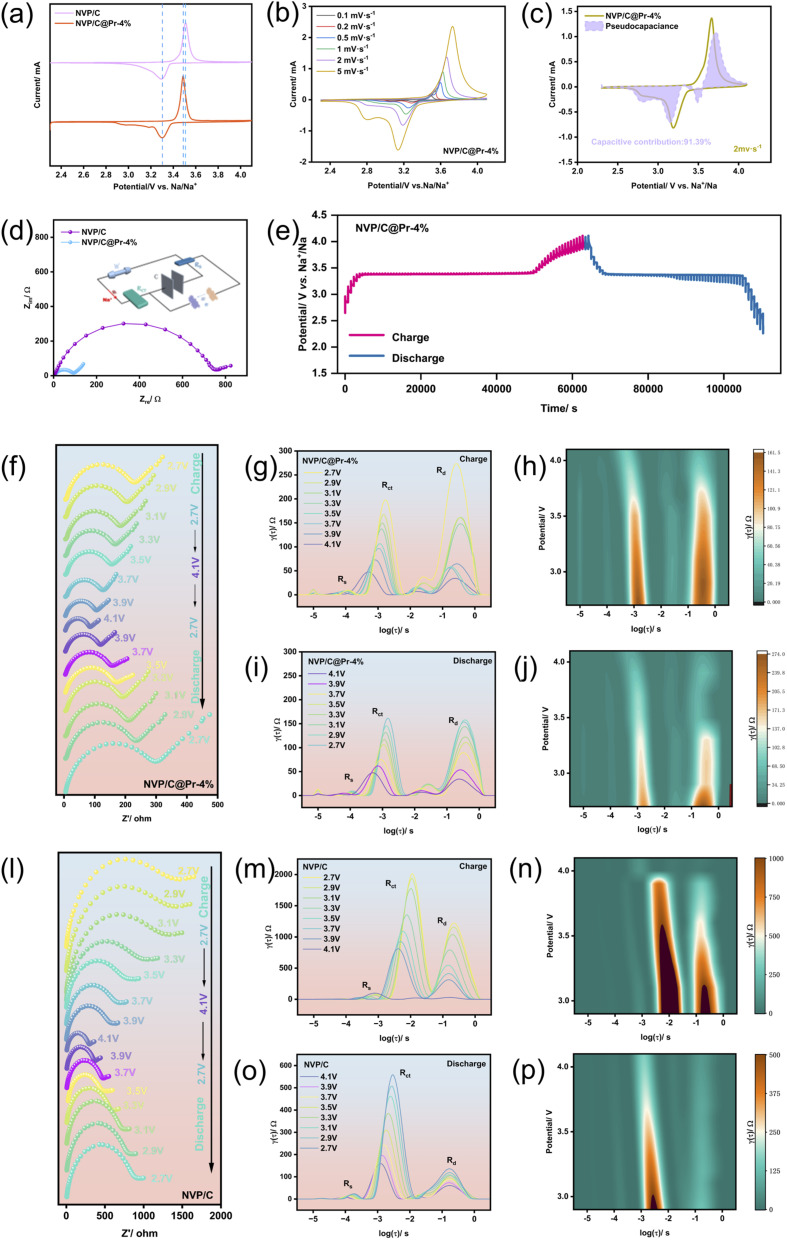
(a) The CV curves of NVP/C and NVP/C@Pr-4% at 0.1 mV s^−1^. (b) CV plots of the NVP/C@Pr-4% sample at different sweeps. (c) The pseudocapacitance control and ion diffusion control of the NVP/C@Pr-4% sample. (d) EIS impedance spectra. (e) The GITT curves depicting the process. *In situ* EIS curves (f), DRT curves of the NVP/C@Pr-4% sample during charging (g) and discharging (i) and their 3D DRT curves (h and j). *In situ* EIS curves (k), DRT curves (l and n) of the NVP sample during charging and discharging and their 3D DRT curves (m and o).

Cyclic voltammetry (CV) is an important technique for studying the electrochemical kinetics of electrode materials. At a scan rate of 0.1 mV s^−1^, the NVP/C@Pr-4% sample at 3.4 V exhibits well-defined symmetrical oxidation and reduction peaks (Fig. 4a), corresponding to the reversible redox reaction of V^3+^/V^4+^. It is worth noting that the NVP/C@Pr-4% sample exhibits smaller voltage polarisation (approximately 0.08 V), which is significantly lower than that of the unmodified NVP/C sample (approximately 0.15 V). This indicates that Pr doping effectively reduces the activation energy barrier of the electrochemical reaction and enhances the electrochemical reversibility of the material. When CV testing was conducted at increased scan rates (0.1–1.0 mV s^−1^) (Fig. 4b), the NVP/C@Pr-4% sample exhibited well-defined CV curves, with the peak current increasing linearly with scan rate. This indicates that the material retains high reversibility and low polarisation even at higher scan rates. According to the Randles–Ševčík equation, the peak current (*I*_p_) exhibits a linear relationship with the square root of the scan rate (*ν*^1/2^), and its slope is directly proportional to the sodium ion diffusion coefficient (*D*_Na^+^_). A linear fit analysis was performed on the NVP/C@Pr-4% sample (Fig. S4b). For NVP/C@Pr-4%, *D*_Na^+^_ = 4.65 × 10^−11^ during charging and *D*_Na^+^_ = 2.39 × 10^−11^ during discharging, which is greater than the diffusion coefficients of NVP/C during charging (*D*_Na^+^_ = 2.58 × 10^−11^) and discharging (*D*_Na^+^_ = 5.39 × 10^−12^) (Fig. S4c). In the equation, the *b*-value can be used to determine the ion transport mechanism of electrode materials. When the *b*-value approaches 0.5, ion transport is dominated by diffusion; when 1 > *b* > 0.5, ion transport is governed by a combination of diffusion and capacitance. The *b*-values were obtained from the slopes (Fig. S4d and e). The results show that the *b*-values of NVP/C@Pr-4% during charge and discharge are 0.66 and 0.85 (Table S2), falling within the range 0.5–1, indicating that the ion transport mechanism involves a combination of diffusion and capacitance. This hybrid transport mechanism endows the material with both diffusion-controlled capacity characteristics and capacitance-controlled rapid kinetic response, enhancing its rate performance. At a scan rate of 2 mV s^−1^, NVP/C@Pr-4% exhibits a faradaic contribution of 91.39% (Fig. 4c), which exceeded by 54.5% that of NVP (Fig. S4f). This high pseudocapacitance contribution is a key factor in the excellent rate capability and cycling stability of the material. Furthermore, electrochemical impedance spectroscopy (EIS) was performed on NVP/C and NVP/C@Pr-4% at 3.7 V. The *R*_ct_ of NVP/C@Pr-4% was 89 Ω, lower than the 754 Ω of NVP/C (Table S3). This improvement is because of the enhanced electrical conductivity resulting from Pr^3+^ ion doping, the formation of a continuous conductive network by the carbon coating, and the reduced ion transport resistance due to optimised crystal structure. The decrease in *R*_ct_ directly reflects the enhanced interfacial charge transfer kinetics, which is conducive to improving the rate capability and cycling stability of the material. Intermittent constant-current titration (GITT) was performed on NVP/C@Pr-4%, yielding charge–discharge curves from which a voltage plateau at 3.4 V was observed, consistent with the charge–discharge plateau obtained from electrochemical measurements. A single titration cycle was selected for the GITT charging and discharging processes of the NVP/C@Pr-4% sample, and linear regression fitting of the data points is shown in Fig. S6. The GITT results indicate that the *D*_Na^+^_ concentration of NVP/C@Pr-4% is of the order of 10^−11^, representing a significant improvement compared to NVP/C, suggesting that the modification strategy has significantly enhanced the diffusion kinetics of sodium ions. Monitoring of the NVP/C@Pr-4% and NVP/C electrodes during the charging and discharging processes using *in situ* electrochemical impedance spectroscopy (Fig. 4f and k) revealed that *R*_ct_ gradually decreased during charging and gradually increased during discharging. For the NVP/C@Pr-4% sample (Fig. 4f), *R*_ct_ gradually decreased during charging and gradually increased during discharging (Fig. 4g and i). This trend is closely related to the insertion and extraction of sodium ions. In the charging process, sodium ions are released from the lattice, causing lattice contraction; the ion transport channels widen, and *R*_ct_ and *R*_d_ decrease. In the discharging process, sodium ions are incorporated into the lattice, causing lattice expansion; the ion transport resistance increases, and *R*_ct_ and *R*_d_ increase. This reversible change in impedance indicates that the material possesses excellent electrochemical reversibility. Fig. 4g, i, l and n show the DRT curves derived from the *in situ* EIS data for NVP/C@Pr-4% and NVP/C, respectively. Three characteristic peaks are present in the curves, representing *R*_s_ (interfacial resistance), *R*_ct_ (charge transfer resistance) and *R*_d_ (ion diffusion resistance). The 3D projections of Fig. 4g and i are shown in Fig. 4h and j; the 3D projections of Fig. 4l and n are shown in Fig. 4m and o. During charging, *R*_ct_ and *R*_d_ gradually decrease, and the peaks shift towards lower relaxation times, indicating that as sodium is extracted, the charge transfer kinetics accelerate and the sodium ion diffusion rate increases. Conversely, the opposite occurs during discharge, demonstrating excellent electrochemical reversibility during cycling. Furthermore, the charge transfer impedance (*R*_ct_) in NVP/C is greater than that in NVP/C@Pr-4%, indicating that Pr doping enhances the conductivity of the material. The ionic diffusion resistance (*R*_d_) decreases, allowing Na^+^ to diffuse more rapidly within the structure.

**Fig. 5:**
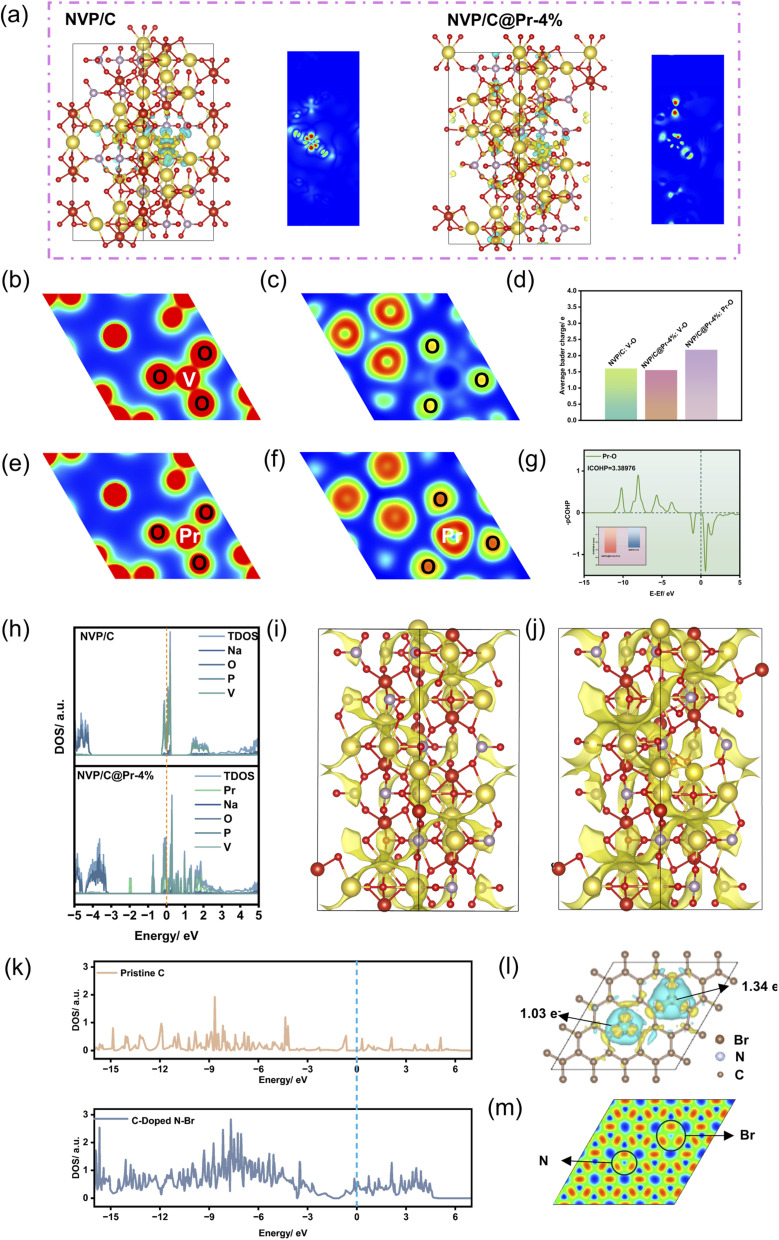
(a) Differential charge density distribution maps of the NVP/C and NVP/C@Pr-4% samples. (b and e) Bader charge profile for the NVP/C and NVP/C@Pr-4% samples. (c and f) Electron localisation function distribution maps for the NVP/C and NVP/C@Pr-4% samples. (d) Comparison of Bader charges between different atoms. (g) Crystal orbital Hamiltonian population integral spectrum (ICOHP spectrum). (h) DOS for the samples. (i and j) Schematic diagrams of the BVEL migration path for Na ions in the samples. (k) DOS analysis of the original carbon layer and the modified carbon layer. (l) Simulated charge density analysis. (m) Partial charge density distributions for the modified carbon layer.

Fig. 5a shows the differential charge density distributions of NVP/C and NVP/C@Pr-4%, illustrating the effect of doping on the electronic structure of the material; yellow indicates regions of electron accumulation, whilst blue indicates regions of electron depletion. It can be observed that in NVP/C@Pr-4%, the yellow regions surrounding the oxygen atoms are more pronounced than in NVP/C, indicating that, following modification, the oxygen atoms have attracted more electrons from the surrounding metal atoms (V and Pr). The density of the electron cloud increases in the overlap region between V and O; this implies that the bonding interactions within the V–O bonds are strengthened. The introduction of Pr alters the overall electrostatic potential, causing the neighbouring V atoms to bind more strongly to O, resulting in the shortening of the V–O bond. Fig. 5b and e show the Bader profile analysis results for NVP/C and NVP/C@Pr-4%, respectively, which provide a quantitative description of the actual charge states of individual atoms within the crystal. In NVP/C, the electron density distribution around V is relatively uniform, and electron transfer occurs primarily within the V–O bonds. In NVP/C@Pr-4%, however, because Pr has a lower electronegativity than V and its 4f electrons are highly localised, the electron density around the O atoms increases, leading to a higher degree of V–O polarisation. Fig. 5c and f show the electron localisation function distributions for NVP/C and NVP/C@Pr-4%, respectively. In NVP/C, the V–O bond exhibits a certain degree of covalency. In NVP/C@Pr-4%, the region between the V and O atoms appears darker than in Fig. 5c, indicating an increase in the covalent character of the V–O bond. This is consistent with the conclusion that Pr doping leads to increased polarisation of the V–O bond and a shortening of the bond length. Fig. 5d illustrates the average charge transfer between different atomic pairs. It is worth noting that the charge transfer on the V–O bond in NVP/C@Pr-4% is slightly lower than that in NVP/C, indicating that the introduction of Pr has altered the electronic environment around V. Fig. 5g presents the integral of the crystal orbital Hamiltonian population (ICOHP). The ICOHP value of −3.38976 for the Pr–O bond indicates that the Pr–O bond is stronger, which is conducive to enhancing the structural stability of the material. Fig. 5h shows the total and partial density of states for NVP/C and NVP/C@Pr-4%. In NVP/C@Pr-4%, the total density of states at the Fermi level is higher than that of NVP/C, indicating that more electrons are involved in conduction, thereby enhancing the electrical conductivity of the material. Following the introduction of Pr, a distinct 4f orbital electronic peak appears to the left of the Fermi level, providing new electronic states.^24^ These hybridise with the V–O bonds, increasing the electron density at the Fermi level, broadening the pathways for electron flow, and enhancing the electrical conductivity of the material. The overlap between the Pr and O peaks indicates strong interaction with the surrounding O atoms, which contributes to the stability of the crystal structure. Fig. 5i and j are schematic diagrams of the BVEL migration path for NVP/C and NVP/C@Pr-4%, respectively. In Fig. 5i, the Na^+^ diffusion channels in NVP/C are narrow, whereas in Fig. 5j for NVP/C@Pr-4%, the pathways are widened, allowing for faster and smoother Na^+^ transport. Compared with the pure carbon layer, N/Br codoping introduces electronic states at the Fermi level, resulting in a continuous band structure. The Br/N-codoped modified carbon layer enhances charge transfer. Fig. 5l shows the differential charge density map of the Br/N-codoped carbon layer, providing a visual representation of the electron transfer.^25^ Bader charge analysis quantitatively demonstrates that Br atoms lose 1.34 e^−^, whilst N atoms gain 1.03 e^−^. As shown in Fig. 5m, the electron localised field (ELF) map reveals that the high electron density around N atoms leads to a weakening of the surrounding C–C bonds, whereas the low electron density around Br atoms results in a strengthening of the C–C bonds. This non-uniform charge distribution promotes charge separation and enhances the chemical reactivity of the carbon layer surface.^26^

**Fig. 6:**
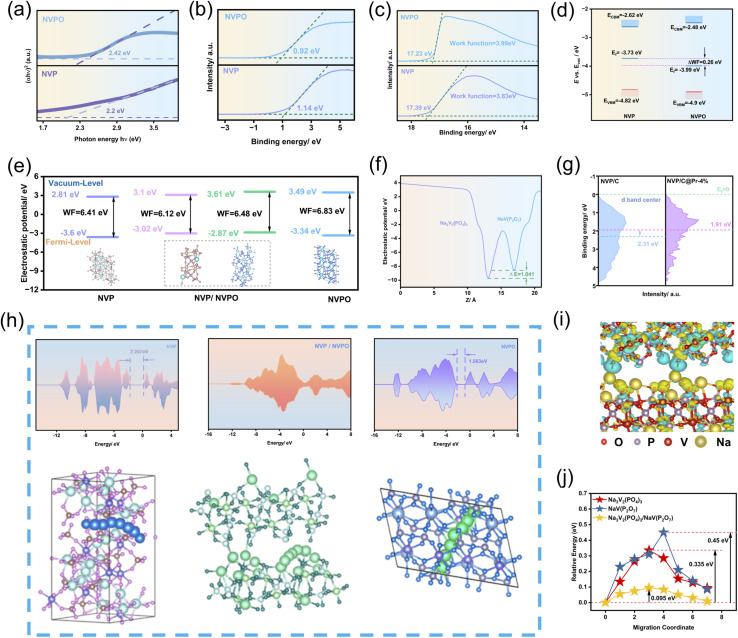
(a) Results of the bandgap test of the sample by UV. (b and c) Results of the intrinsic work function test of the sample by UPS. (d) Energy band gradient diagram of the samples. (e) Theoretical simulation results for NVP, NVPO and composite phase work function. (f) Simulation results of electrostatic energy potential. (g) D-band center of the samples. (h) TDOS and simulation of sodium ion migration pathways of NVP, NVPO, and NVP/NVPO. (i) Charge distribution in NVP/NVPO systems. (j) Calculation of the energy barriers for sodium ion migration in various phases.

When dissimilar materials with different work functions come into contact, the interface charges undergo spontaneous reorganisation, generating an intrinsic electric field.^27^ This intrinsic electric field provides a high-speed transport pathway for charge transfer, significantly enhancing the kinetics of electron and ion transport. As determined by ultraviolet-visible diffuse reflectance spectroscopy (UV-vis), the bandgap widths of the pure NVPO and NVP phases were 2.42 eV and 2.2 eV, respectively (Fig. 6a). The intrinsic work functions of the pure-phase NVPO and NVP were further analysed using ultraviolet-visible photoelectron spectroscopy (UPS), with the results showing that the work functions of the pure NVP and NVPO phases were 3.83 eV and 3.99 eV, respectively (Fig. 6b and c). Fig. 6d shows the band structures of NVP and NVPO; the difference in band energy levels between the two materials gives rise to an intrinsic electric field at the interface. First-principles calculations indicate that the work function of pure NVP is 6.41 eV, whereas that of pure NVPO is 6.83 eV; in the NVP/NVPO composite phase, the work function of NVP decreases to 6.12 eV and that of NVPO to 6.48 eV, both of which are lower than the work function values of the pure materials. The presence of an electrostatic barrier contributes to the formation of an intrinsic electric field (Fig. 6f); an electrostatic barrier of 1.041 eV was observed in the NVP/NVPO composite phase. Analysis of the XPS valence band spectrum (Fig. 6g) reveals that the D-band centre of NVP/C@Pr-4% shifts towards the Fermi level, indicating that electrons are more readily excited, enhancing the electrical conductivity of the material. This further confirms the presence of the built-in electric field and its role in promoting electrical conductivity. Fig. 6h shows the total density of states (TDOS) calculated for NVP, NVP/NVPO and NVPO. It can be seen that NVP has a bandgap of 2.257 eV, the NVP/NVPO two-phase heterostructure has no bandgap, and NVPO has a bandgap of 1.563 eV. This indicates that the heterostructure exhibits properties similar to those of a metal, with reduced electronic transport resistance, which is conducive to enhancing the intrinsic conductivity of the polycation. First-principles calculations were employed to investigate the charge distribution at the two-phase interface; Fig. 6i shows the molecular structural model of the NVP/NVPO two-phase interface. In the pure phases, the charge distribution is sparse, whereas at the two-phase interface, the charge density is high. Calculations of the sodium ion migration energy barrier (Fig. 6j) indicate that the sodium ion migration energy barrier is 0.335 eV in NVP and 0.45 eV in NVPO, whereas it decreases to 0.095 eV at the NVP/NVPO heterojunction interface. This suggests that the heterojunction interface significantly reduces the transport resistance of sodium ions, optimising the electrochemical performance of the material. These results indicate that the NVP/NVPO heterostructure significantly improves the electrochemical performance of the material through mechanisms such as the formation of an intrinsic electric field, optimisation of the electronic structure, and reduction of the ionic migration energy barrier.

**Fig. 7:**
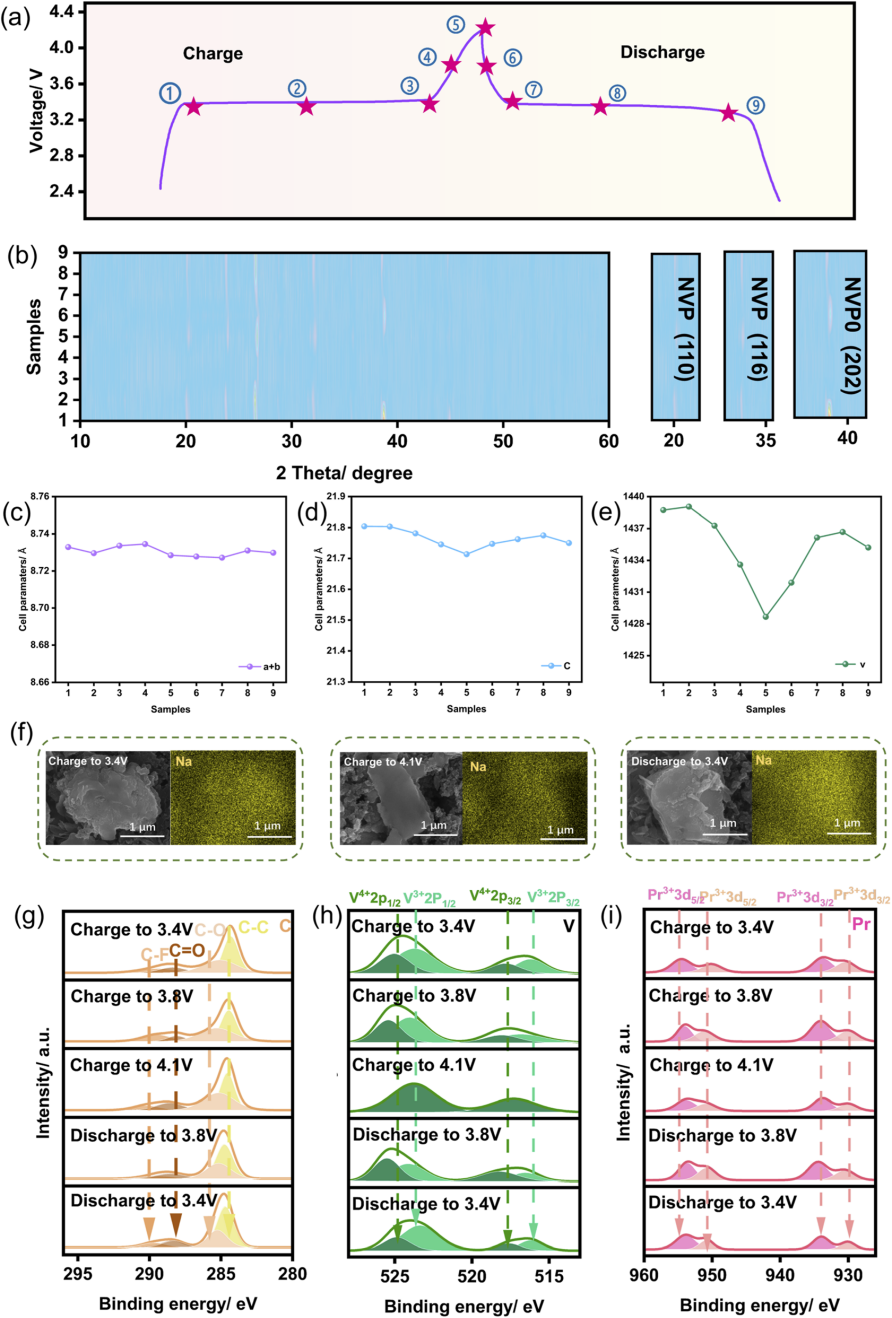
(a) GCD curve of the NVP/C@Pr-4% sample. (b) XRD curves of the NVP/C@Pr-4% sample at different voltages and local enlargements. (c–e) *Ex situ* XRD refinement parameters. (f) Mapping plots of sodium at different voltages. *Ex situ* XPS of C (g), V (h), Pr (i) at different voltage sites.

To investigate the sodium-ion storage mechanism of NVP/C@Pr-4% during charge–discharge cycling, the electrode material was systematically characterised using *in situ* X-ray diffraction (XRD) techniques.^28^ As shown in Fig. 7a, nine key states were selected from a single charge–discharge cycle for *in situ* sampling. Fig. 7b presents the XRD patterns of NVP/C@Pr-4% in various states (compared with the standard NVP structure for reference). The magnified views clearly indicate that the characteristic diffraction peaks corresponding to the (110) and (116) crystal planes of the NVP phase, as well as the (202) crystal plane of the NVPO phase, show virtually no shift throughout the complete charge–discharge cycle, demonstrating a high degree of structural reversibility. Fig. 7c–e quantitatively illustrate the evolution of the unit cell parameters during this process (Table S4). Analysis reveals that the *a*-axis parameter undergoes only slight changes, whereas the expansion and contraction of the *c*-axis show a high degree of consistency with the overall change in unit cell volume; this confirms that sodium ions undergo reversible intercalation and deintercalation primarily along the *c*-axis during charging and discharging. Elemental analysis of NVP/C@Pr-4% under three states—charged to 3.4 V, charged to 4.1 V, and discharged to 3.4 V—reveals that the Na content decreases upon charging to 4.1 V, and then increases upon discharging to 3.4 V. Further investigation using *ex situ* X-ray photoelectron spectroscopy (XPS) was conducted to examine the evolution of the chemical states of the three elements C, V and Pr, at five key voltage points (charging to 3.4 V, 3.8 V and 4.1 V, and discharging to 3.8 V and 3.4 V) (Fig. 7g–i). The C 1s fine-structure spectrum exhibited no significant shifts or changes in peak shape across all states, indicating that the surface carbon coating remains structurally stable during the complex sodium deintercalation and intercalation processes, endowing the material with excellent interfacial stability. The V 2p spectrum, however, clearly reveals the microscopic processes of electrochemical redox reactions: during charging from 3.4 V to 4.1 V, the relative content of V^3+^ gradually decreases, whereas that of V^4+^ correspondingly increases. By 4.1 V, V^3+^ is almost completely oxidised to V^4+^. During the subsequent discharge process, V^4+^ is gradually reduced back to V^3+^. This result provides direct evidence that the charge–discharge process is dominated by the highly reversible V^3+^/V^4+^ redox pair. In stark contrast, the Pr 3d spectrum remains stable throughout the entire charge–discharge cycle, with no change in valence state. This not only reaffirms that the Pr element does not participate in electrochemical redox reactions but also indicates that Pr^3+^ acts as a structurally rigid pillar within the lattice, effectively maintaining the structural integrity of the material.

**Fig. 8:**
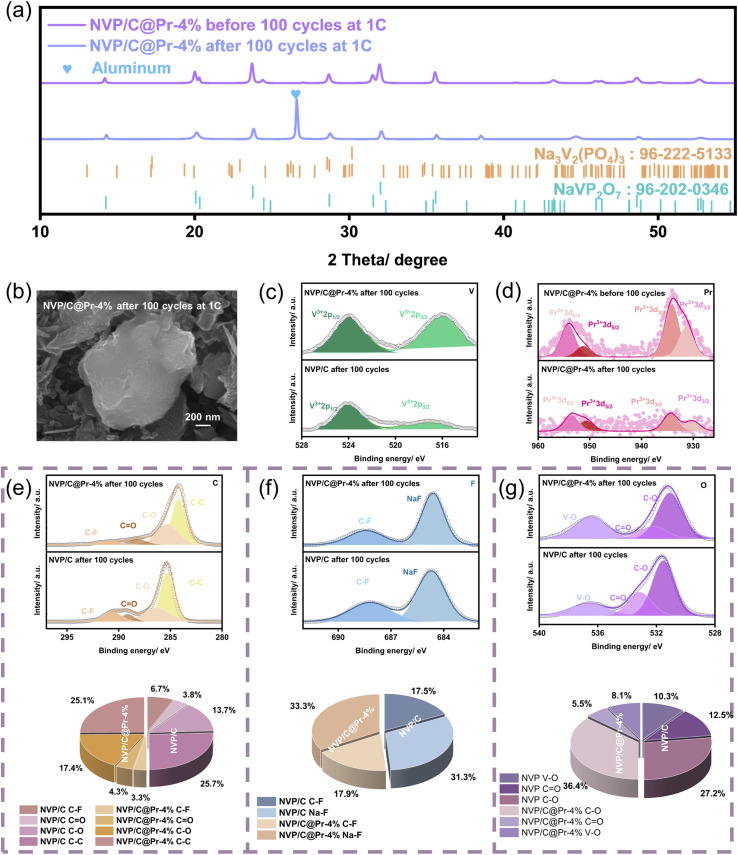
(a) XRD images before and after cycling, (b) SEM image of the NVP/C@Pr-4% sample after 100 cycles. XPS images of V (c), Pr (d), C (e), F (f) and O (g) in the NVP/C@Pr-4% sample and the NVP sample after cycling.

To evaluate the cycling stability of the NVP/C@Pr-4% material, a series of post-cycling characterisation tests were conducted. Fig. 8a shows the X-ray diffraction (XRD) patterns of NVP/C@Pr-4% before and after 100 cycles.^29^ It can be seen that the positions of the diffraction peaks before and after cycling closely coincide, indicating that the material retains good crystallinity and structural stability even after prolonged cycling. Scanning electron microscopy (SEM) images of the electrode sheets after cycling (Fig. 8b) reveal that the particles remain uniformly dispersed after 100 cycles. This indicates that the rigid NASICON framework effectively suppresses volume expansion during charging and discharging, endowing the material with excellent structural stability. Furthermore, no obvious cracks or pores were observed on the electrode surface, further confirming the outstanding cycling stability of NVP/C@Pr-4%.^30^ To gain a deeper understanding of the mechanisms underlying the interfacial evolution and structural stability of NVP/C@Pr-4% during long-term cycling, a comparative X-ray photoelectron spectroscopy (XPS) analysis was conducted between the post-cycling NVP/C@Pr-4% electrode and the unmodified NVP/C electrode (Fig. 8c). In the V 2p fine-structure spectrum, no significant shift in the binding energy position of V^2+^ was observed in either sample; however, the peak intensities exhibited a marked difference: the vanadium signal intensity in NVP/C@Pr-4% was significantly higher than that in NVP/C. This phenomenon strongly demonstrates that the introduction of Pr^3+^ ions acts as a “rigid scaffold”, anchoring the crystal lattice through strong bonding interactions. This significantly suppresses the severe dissolution of vanadium caused by phase transitions or electrolyte corrosion during cycling, allowing the modified electrode surface to retain a high concentration of active vanadium species, fundamentally slowing down the loss of active material and capacity decay. Furthermore, observation of the Pr 3d spectrum reveals a significant attenuation in the intensity of its characteristic peaks following cycling. This is not attributable to the dissolution of Pr during the electrochemical process, but rather to the *in situ* formation of a dense cathodic electrolyte interface (CEI) film on the electrode surface during charging and discharging.^31^ This coating exerts a physical shielding and photoelectric attenuation effect on the Pr signal originating from the bulk interior.^32^ It is worth noting that, despite the reduced peak intensity, the binding energy of Pr did not undergo any discernible shift. This conclusively indicates that the valence state of Pr remained firmly at +3 throughout the entire electrochemical process, and its local chemical environment remained intact. This high degree of electrochemical inertness implies that Pr doping did not induce any irreversible side reactions; rather, it continued to stabilise the crystal framework, maintaining the intrinsic electrochemical activity of the material. A detailed analysis of the interfacial chemical environment was conducted by combining the detailed spectra of C 1s (Fig. 8e), F 1s (Fig. 8f) and O 1s (Fig. 8g). The spectra reveal slight shifts in the positions of the characteristic peaks following cycling, reflecting the dynamic adjustment of the interfacial electronic states driven by electrochemical processes. Notably, in the C 1s spectra after 100 cycles, the two samples exhibited distinctly different interfacial compositions: on the surface of the unmodified NVP/C electrode, the C–C peak—representing the conductive carbon network framework—diminished sharply, whilst the peaks corresponding to oxygen-containing organic compounds (C–O, C=O) were exceptionally prominent. This indicates that in NVP/C, the electrolyte undergoes severe oxidative decomposition under high voltage, resulting in the uncontrolled deposition of a large amount of liquid organic by-products (such as polycarbonates) on the electrode surface, forming a CEI layer with complex composition and high impedance. In contrast, the NVP/C@Pr-4% sample, benefiting from the surface modification effect of Pr, effectively modulated the electronic structure at the interface, significantly suppressed the excessive oxidation of electrolyte solvent molecules, and guided their directional decomposition, promoting the formation of a highly stable CEI film. This uniform and dense CEI film acts as a ‘protective shield’, blocking the continuous erosion of active material by the electrolyte. At the same time, the F 1s spectrum revealed that, compared to NVP/C, the intensity of the characteristic peak corresponding to the primary C–F bond (derived primarily from the PVDF binder or electrolyte salts) was significantly reduced in NVP/C@Pr-4%. This transformation reveals that, driven by electrochemical cycling, some C–F bonds break and participate in interfacial restructuring, converting *in situ* into a CEI film rich in inorganic NaF upon binding with Na^+^. In sodium-ion battery systems, NaF is regarded as one of the most ideal inorganic components of the CEI due to its high dielectric constant and high mechanical strength. This NaF-rich interfacial layer effectively accommodates the volumetric strain during charging and discharging, suppressing the collapse and fragmentation of the crystal structure on the surface of the material, and significantly reducing the rate of subsequent interfacial side reactions. Furthermore, the evolution of the O 1s XPS spectrum is highly consistent with the above conclusions, providing further confirmation from the perspective of the oxygen coordination environment of the exceptional protective efficacy of Pr doping on the crystal structure of both the bulk and surface of the material. In summary, this high-quality CEI layer—optimised in composition and structurally stable—induced by Pr modification not only eliminates harmful side reactions but also provides a low-impedance transport pathway for the rapid deintercalation of Na^+^, representing one of the core mechanisms for achieving long-term cycling stability of the electrode.^32^

**Fig. 9:**
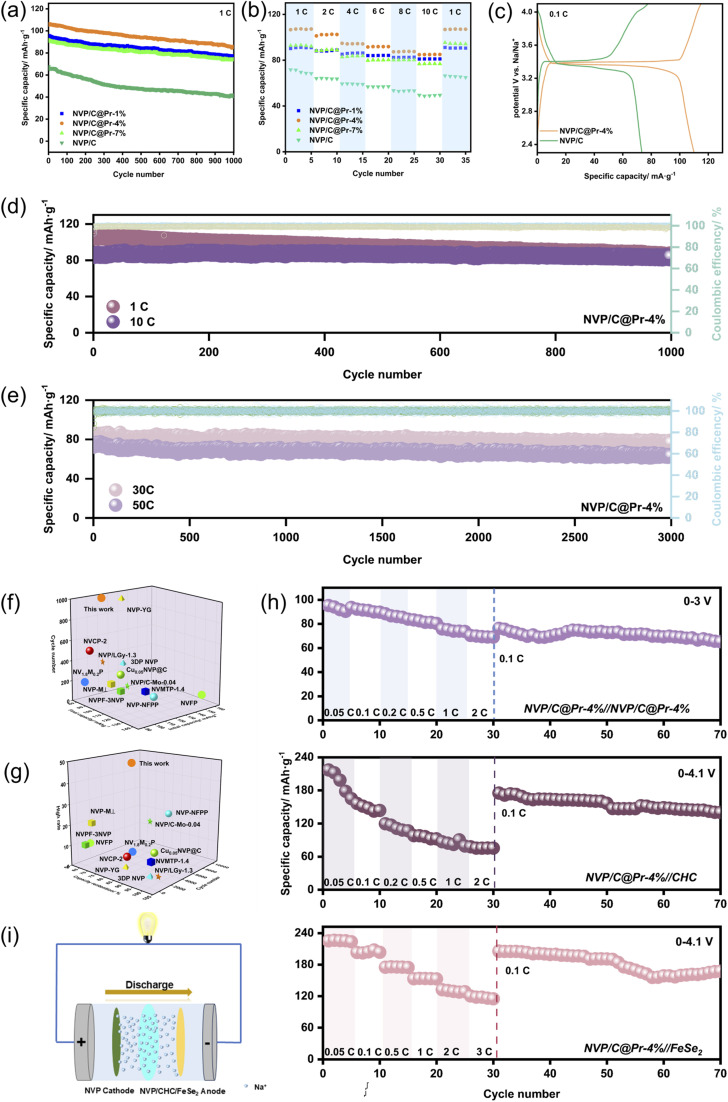
(a) Cycle curves of all samples at 1C. (b) Curves of all samples at different magnifications. (c) First curves of GCD of NVP/C and NVP/C@Pr-4% at 0.1C. Long cycling curves for the NVP/C@Pr-4% sample at 1C and 10C (d), 30C and 50C (e). (f and g) Comparison of performance between previous studies and this experiment. (i) Diagram of the battery. (h) NVP/C@Pr-4% generalizability test.

To systematically evaluate the synergistic effect of Pr doping and carbon coating on the improvement in the electrochemical performance of the material, the prepared samples were fabricated into electrodes and assembled into half-cells for testing. As shown in Fig. 9a, long-term cycling test results at a current density of 1C (1C ≈ 118 mAh g^−1^) indicate that NVP/C@Pr-4% exhibits the best sodium storage capacity, with an initial discharge specific capacity as high as 105.4 mAh g^−1^; even after 1000 charge–discharge cycles, the capacity remained at 85.13 mAh g^−1^, corresponding to a capacity retention rate of 81.02%. This performance is significantly superior to that of NVP/C@Pr-1% (95.4 mAh g^−1^), NVP/C@Pr-7% (91.2 mAh g^−1^) or unmodified NVP/C (67.7 mAh g^−1^). These results not only confirm the effectiveness of moderate Pr doping, but also suggest that excessive doping (*e.g.* NVP/C@Pr-7%) may compromise lattice integrity, inhibiting electrochemical performance. Subsequently, the four samples were subjected to performance testing at varying magnifications (1 C–10C, Fig. 9b). At each test rate, the specific capacities for NVP/C@Pr-4% were 106.6, 101.2, 94.7, 91.5, 87.3 and 84.8 mAh g^−1^, respectively; furthermore, upon returning to a low rate following high-rate testing, the capacity recovered to 106.8 mAh g^−1^. All these metrics significantly outperformed those of the NVP/C sample. A detailed analysis of the constant-current charge–discharge (GCD) curves in Fig. 9c reveals that NVP/C@Pr-4% exhibits a broader, longer and flatter charge–discharge voltage plateau, which clearly reflects its higher contribution to the actual specific capacity. More crucially, the voltage gap (polarisation) between the charge and discharge platforms of NVP/C@Pr-4% was significantly reduced, confirming that the synergistic effect of Pr doping and the heterostructure effectively lowered the charge transfer barrier, endowing the material with extremely fast sodium-ion intercalation and deintercalation kinetics. To further investigate the cycling stability of the material at high current rates, cycling evaluations were conducted on NVP/C@Pr-4% under ultra-high current conditions (Fig. 9d and e). After 1000 cycles at a high current of 10C, the capacity showed virtually no decline (remaining at 83.3 mAh g^−1^); notably, after 3000 cycles at an ultra-high current density of 30C, the capacity retention rate remained as high as 99.31%, demonstrating long-term potential for ultra-fast charging and discharging; even under test conditions of 50C, it could still deliver a capacity of 73.8 mAh g^−1^ and maintain a retention rate of 86.13%. This breakthrough in high-rate performance and long-term stability can be attributed primarily to the built-in electric field, as described earlier, which accelerates electron/ion transport, and the rigid framework, which effectively resists high-frequency volumetric strain. Compared with sodium–vanadate–phosphate-based cathode materials reported in recent mainstream literature (Fig. 9f and g), NVP/C@Pr-4% demonstrates outstanding advantages in comprehensive electrochemical metrics^33–44^ (Table S5). Given the differences between laboratory half-cell testing and actual commercial applications, the performance evaluation of full cells is of paramount importance. To this end, full-cell systems were assembled using NVP/C@Pr-4% as the cathode, paired with either commercial hard carbon (CHC) or commercial iron selenide (FeSe_2_) as the anode (Fig. 9h). To compensate for the irreversible sodium ion consumption by the anode during the first charge–discharge cycle, both CHC and FeSe_2_ underwent rigorous pre-sodiation treatment prior to assembly. At a low rate of 0.05C, the NVP/C@Pr-4%‖CHC and NVP/C@Pr-4%‖FeSe_2_ full-cell systems exhibited discharge capacities of 217 mAh g^−1^ and 225.2 mAh g^−1^, respectively; even at a rate of 2C, they maintained capacities of 77.7 mAh g^−1^ and 132.2 mAh g^−1^. Furthermore, when the rate was restored to 0.1C, the discharge capacities effectively recovered to 76.3, 175.2 and 205.2 mAh g^−1^, providing preliminary validation of the structural reversibility of the full-cell system. Fig. 9i provides a clear illustration of the detailed electrochemical behaviour of the NVP/C@Pr-4%‖CHC full cell. In stepwise rate tests from 0.05C to 2C, the specific discharge capacities were 217.2, 178.4, 119.5, 98, 86.8 and 77.7 mAh g^−1^, respectively. Upon completion of the high-rate testing and return to a 0.1C rate, the capacity stabilised at 175.2 mAh g^−1^, with no significant capacity decay observed, demonstrating excellent interface stability and reaction reversibility. Furthermore, after 40 continuous cycles, the full cell still maintained a capacity of 140.1 mAh g^−1^. This series of full-cell data fully confirms that the NVP/C@Pr-4% cathode material not only possesses outstanding intrinsic electrochemical activity, but also exhibits good compatibility with existing commercial anodes, demonstrating excellent prospects for practical application.

## 4. Conclusion

In this experiment, the solvothermal method was employed to successfully synthesize a sodium vanadate phosphate composite cathode material (NVP/C@Pr-4%). This material not only exhibits bulk rare-earth doping but also features the formation of a second phase at the reduction site, as well as a carbon layer codoped with heteroatoms. Experiments and theoretical calculations indicate that the built-in electric field at the heterojunction in the NVP/C@Pr-4% material is conducive to sodium ion transport. Furthermore, the synergistic modification of the carbon layer with Br and N reduces interfacial charge transfer resistance and enhances the wettability of the electrode and electrolyte, endowing the NVP/C@Pr-4% cathode material with excellent electrochemical performance. Full-cell testing indicates that the material has practical application value and promising prospects.

## Author contributions

Yanru Huo: investigation, writing – original draft. Shuli Li: data curation. Mengxian Zhang: resources. Xu Yang: formal analysis. Siyuan Li: visualization. Yu Feng: methodology. Hongen Shi: conceptualization. Shanghong Duan (corresponding author): methodology, supervision. Yanjun Chen (corresponding author): funding acquisition, project administration, supervision, writing – review and editing.

## Conflicts of interest

The authors declare no conflicts of interest.

## Supplementary Material

SC-OLF-D6SC05330K-s001

## Data Availability

The data supporting this article have been included as part of the supplementary information (SI). Supplementary information: materials preparation, characterization, electrochemical measurement, calculation details, analysis of the electron gap based on UV-Vis diffuse reflectance spectra. See DOI: https://doi.org/10.1039/d6sc05330k.
