# Supplementary material for: Orbital hybridization engineering, a built-in electric field, and simultaneous carbon layer charge rearrangement boost carrier migration for a superior cathode material
Source: Chem Sci. 2026 Aug 11;17(38):18248–66. doi: 10.1039/d6sc05330k (PMC13458971; doi:10.1039/d6sc05330k)
Supplement: Supplementary file 1 [file SC-017-D6SC05330K-s001.pdf]

Supplementary Information (SI) for Chemical Science.
This journal is © The Royal Society of Chemistry 2026

Supporting Information

Orbital hybridization engineering and built-in electric field simultaneously with carbon layer charge rearrangement boosting carrier migration for superior cathode material

2 Experiment

2.1 Materials preparation

NVP/C@Pr-x% (x = 1, 4, 7) samples were synthesised using the solvothermal method. In this method, $C_6H_{12}O_6 \cdot H_2O$ (Aladdin, 99.5%), $NH_4H_2PO_4$ (Aladdin, 99.0%), $Na_2CO_3 \cdot H_2O$ (Aladdin, 99.99%), V_2O_5 (Aladdin, 99.5%) and Pr $(NO_3)_3 \cdot 6H_2O$ (Aladdin, 99.9%) as starting materials. The sample preparation quantity is 3 g. First, a specified amount of CH_3OH (Aladdin, 99.8%) is used as the solvent, followed by the addition of V_2O_5 in a stoichiometric ratio and an excess of $C_6H_{12}O_6 \cdot H_2O$. The mixture is stirred thoroughly at 80 °C in a constant-temperature water bath at 400 rpm to ensure complete reduction of V. Next, add equimolar amounts of $NH_4H_2PO_4$, $Na_2CO_3 \cdot H_2O$ and Pr $(NO_3)_3 \cdot 6H_2O$ to the above solution, and stir continuously in a constant-temperature water bath at 80 °C and 400 rpm until 20–30 ml of solution remains. Add 2 ml of polyethylene glycol (PEG, Aladdin, average Mn 200) and 10 wt% Cetyltrimethyl Ammonium bromide (CTAB, Aladdin,99%) to the dust-free hydrothermal reactor, then add the remaining solution to the hydrothermal reactor. The hydrothermal reactor was placed in an oven and reacted at 180 °C for 24 hours, then dried at 80 °C to obtain the precursor powder. The powder was placed in a tube furnace and calcined at 450 °C for 4 hours under a nitrogen atmosphere, followed by sintering

at 700 °C for 6 hours, thereby producing the samples NVP/C@Pr-x% (NVP/C@Pr-1%, NVP/C@Pr-4%, NVP/C@Pr-7%).

2.2 Characterization

The crystal structure of the samples was determined by X-ray diffraction (XRD, Bruker AXS D8), with a scanning range of 10°–80° and a scanning speed of 2°/min. The morphology of the NVP/C@Pr-x% (x = 1, 4, 7) powders was examined using a scanning electron microscope (SEM, HITACHI SU8010) and a transmission electron microscope (TEM, Titan G260-300). The binding energies of sodium, vanadium, oxygen, carbon, bromine and praseodymium were analysed using an X-ray photoelectron spectrometer (XPS, Thermo Scientific K-Alpha). The Raman spectra of carbon were verified using a Raman spectrometer (Raman, HORIBA Scientific LabRAM HR Evolution). Thermogravimetric analysis (TGA, Mettler-Toledo) was employed to determine the carbon mass percentage of the NVP/C@Pr-4% samples. Oxygen vacancy signals were detected by electron paramagnetic resonance (EPR, Bruker EMXplus). The Fourier transform infrared (FT-IR) spectra of NVP and NVP/C@Pr-4% were acquired using a Bruker Vertex 70 spectrophotometer, with a wavenumber range of 1500 cm^{-1} to 500 cm^{-1}. X-ray absorption spectroscopy (XAS) analysis was performed at the Beijing Synchrotron Radiation Facility. The L3-edge XANES and EXAFS spectra of V were measured at the 1W1B beamline of the National Synchrotron Radiation Research Centre (NSRRC) in Beijing, China. The L3-edge XAFS data for V were recorded in transmission mode and collected under ambient conditions. V foil, V_2O_3, VO_2, and V_2O_5 were used as reference samples. All XAS

spectra were processed using the DEMETER software package. Following normalisation, the data were analysed using the Athena and Artemis software packages.

Data processing, data analysis and EXAFS fitting were performed using the Athena and Artemis programmes within the Demeter data analysis package; this package utilises the FEFF6 programme for fitting the EXAFS data. Energy calibration of the samples was carried out using a standard vanadium foil (V foil), which was measured simultaneously as a reference sample. A linear function was subtracted from the pre-edge region, followed by normalisation of the edge jump using the Athena software. The $\chi(k)$ data were separated by subtracting a smooth cubic polynomial (which approximates the absorption background of isolated atoms). Following the application of a Hanning window function ($\Delta k = 1.0$), a Fourier transform was performed on the k^3-weighted $\chi(k)$ data. For EXAFS modelling, the Fourier-transformed data in R-space were subjected to a non-linear least-squares fit of the EXAFS equation using Artemis software, thereby obtaining the global amplitude EXAFS parameters (CN, R, σ^2 and ΔE_0). For the wavelet transform analysis, the $\chi(k)$ data exported from Athena were input into the Hama Fortran code.

2.3 Electrochemical measurement

The electrochemical performance of NVP/C@Pr-x% was evaluated using CR2025 half-cells assembled in an argon-filled glove box. The cathode material consisted of active material, acetylene carbon black and polyvinylidene fluoride (PVDF) in a mass ratio of 7:2:1. The raw materials were added to a ball milling jar in the specified proportions and milled for 5 hours in a planetary ball mill to prepare the slurry. The

slurry was then coated onto suitable aluminium foil and vacuum-dried at 120 °C for 12 hours to produce the final cathode. The resulting electrode was punched into 16 mm diameter discs with an average loading density of 1.5 mg cm^{-2}. The electrolyte used was a 1 M $NaClO_4$ solution in poly (propylene carbonate) (PC), containing 2% by volume of fluoroethylene (FEC). A Celgard 2400 membrane was selected as the separator. A circular sodium metal block with a diameter of 14 mm and a thickness of 1.4 mm was used as the cathode. Half-cell cyclic voltammetry and electrochemical impedance spectroscopy (EIS) tests were performed using an electrochemical workstation (AUIVIUM). Cyclic voltammetry was conducted over a voltage range of 2.3 to 4.1 V at scan rates of 0.1, 0.2, 0.5, 1, 2 and 5 mV s^{-1}. Electrochemical impedance spectroscopy (EIS) analysis was performed over a frequency range of 0.01 Hz to 100 kHz, using an AC amplitude of 10 mV. In the LAND battery system, constant-current charge-discharge measurements and constant-current intermittent titration (GITT) tests were conducted within the specified voltage window. The synthesised cathode sheets were then paired with commercial hard carbon (CHC) and assembled into complete cells for electrochemical testing. The mass ratio of the mixture of commercial hard carbon and ultra-P to carboxymethyl cellulose (CMC) (which contains conductive carbon black and sodium) is 8:1:1. The uniformly mixed anode slurry is evenly coated onto copper foil and dried at the aforementioned temperature. The copper foil was cut to one-quarter the size of an A4 sheet and subsequently encapsulated with a sodium tab to form a CR2025 half-cell. Two charge-discharge cycles were performed at a rate of 0.1C to complete the pre-sodation treatment. Upon completion, the assembled cell was

removed from the glove box, and the pre-sodated anode sheet was immediately removed. Subsequently, the positive and negative electrodes, separator and electrolyte were encapsulated to form a complete CR2025 battery, and constant-current charge-discharge testing was conducted. The FeSe anode used to test the suitability of the anode in this experiment was prepared using the same method as described above, with the sole substitution of commercial hard carbon (CHC) with FeSe.

2.4 Calculation details

The calculations were carried out using the Vienna Ab initio Simulation Package (VASP) based on the plane - wave pseudopotential method. The projector - augmented wave (PAW) method was employed to describe the electron - ion interactions. [Phys. Rev. B 1994, 50, 17953; Phys. Rev. B 1999, 59, 1758] The generalized gradient approximation (GGA) in the form of the Perdew - Burke - Ernzerhof (PBE) functional [Phys. Rev. Lett. 1996, 77, 3865] was used to treat the exchange - correlation energy. To account for the strong electron - electron correlation effects in vanadium (V), the DFT+U approach was adopted. Specifically, a Hubbard U parameter of U=3.25 eV was set for V atoms. Before the calculation of the Li - ion migration energy barrier, the initial and final structures of the system were fully optimized. The optimization was performed with a plane wave cutoff energy of 500 eV. The The Monkhorst - Pack k point mesh [8] with a density of $3\times3\times3$ was used for sampling the Brillouin zone. The convergence criteria for the self - consistent field (SCF) iteration was set to an energy change of less than 10-5 eV between consecutive steps, and the maximum force on each atom was required to be less than 0.05 eV/Å. The work function changes have been calculated for different configurations. The electrostatic potential Vr was the output from VASP without exchange-correlation potential (LVHAR=.TRUE.) by equation as follows:

$$Z_r = \sum_A \frac{Z_A}{|R_A - r|} - \int \frac{\rho(r')dr}{|r' - r|}$$

For nucleus A, the charge, ZA, and position, RA, are defined. Meanwhile, r and ρ(r) represent the spatial coordinate and the electronic density function, respectively.

The CINEB method [J. Chem. Phys. 2000, 113, 9901-9904; J. Chem. Phys. 2000, 9978-9985] implemented in VASP was used to calculate the minimum energy path (MEP) and the corresponding energy barrier for the Li - ion migration within the material.

2.5 Analysis of the electron gap based on UV-Vis diffuse reflectance spectra.

Calculation method for Tauc plots derived from UV-Vis diffuse reflectance spectra:

For semiconductor materials, the following relationship holds between the bandgap and the absorption coefficient:

$$(\alpha h\nu)^{\frac{1}{n}} = B \times (h\nu - Eg)$$

where α is the absorption coefficient, hν is the photon energy, and h is Planck's constant constant (h≈4.13567×10-15 eV·s), ν is the incident photon frequency (ν=c/λ,where c is the speed of light, c≈3×10^8 m/s; λ is the wavelength of the incident light), B is the proportionality constant, and Eg is the forbidden-band width of the semiconductor material.

3 Result data

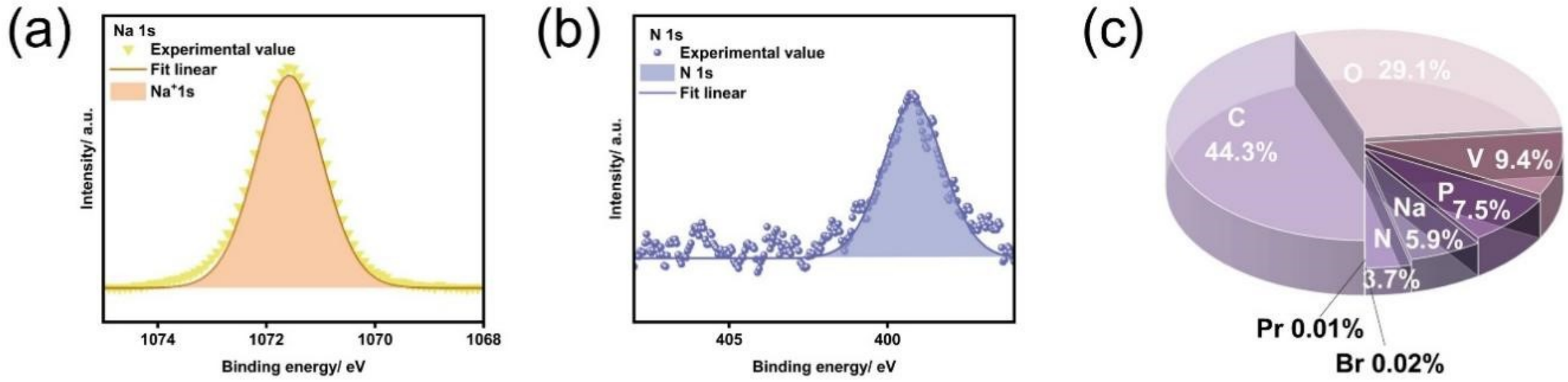

Figure S1 XPS energy spectrum of elements (a) Na (b) N (c) The percentage of each element in NVP/C@Pr-4%.

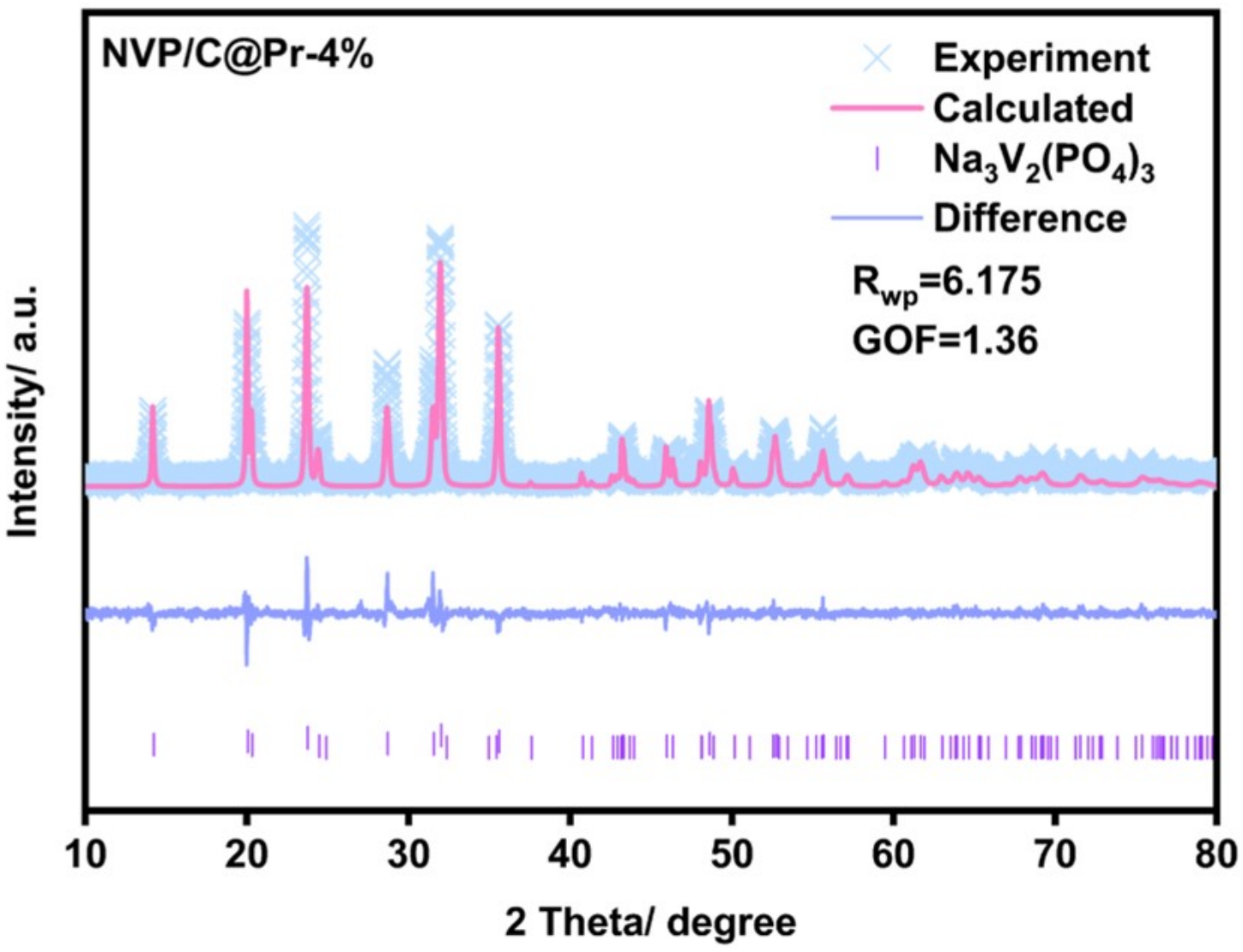

S

Figure S2 Rietveld refined result of NVP/C@Pr-4% by GSASII program

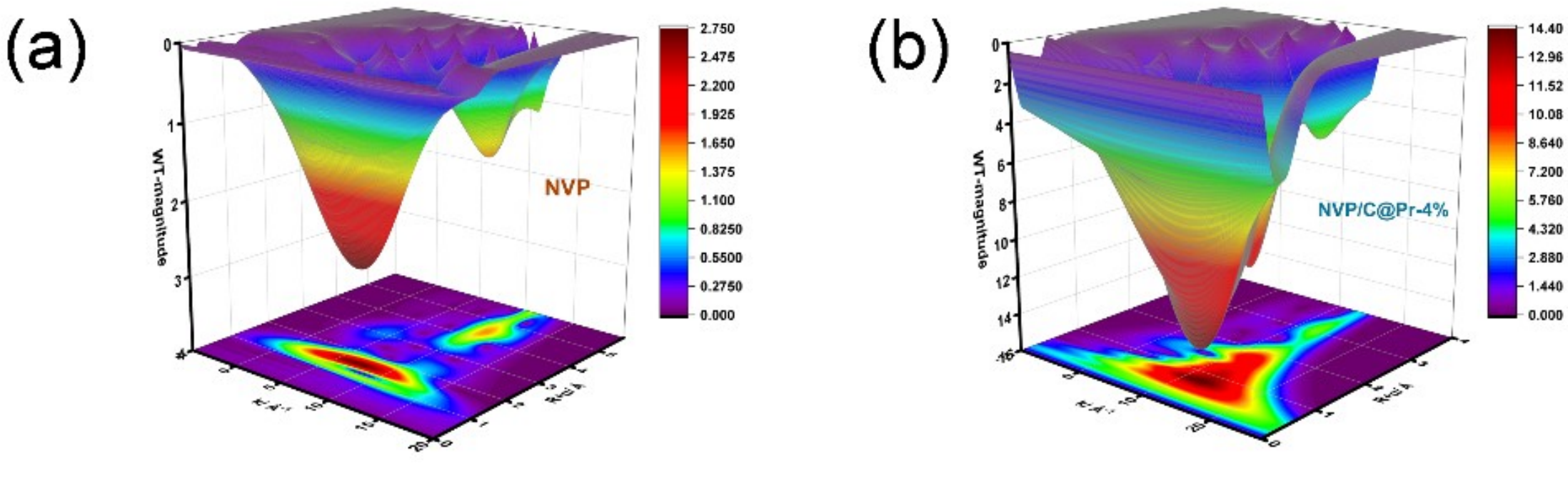

Figure S3 Wavelet transform 3D plot of (a)NVP/C(b)NVP/C@Pr-4%

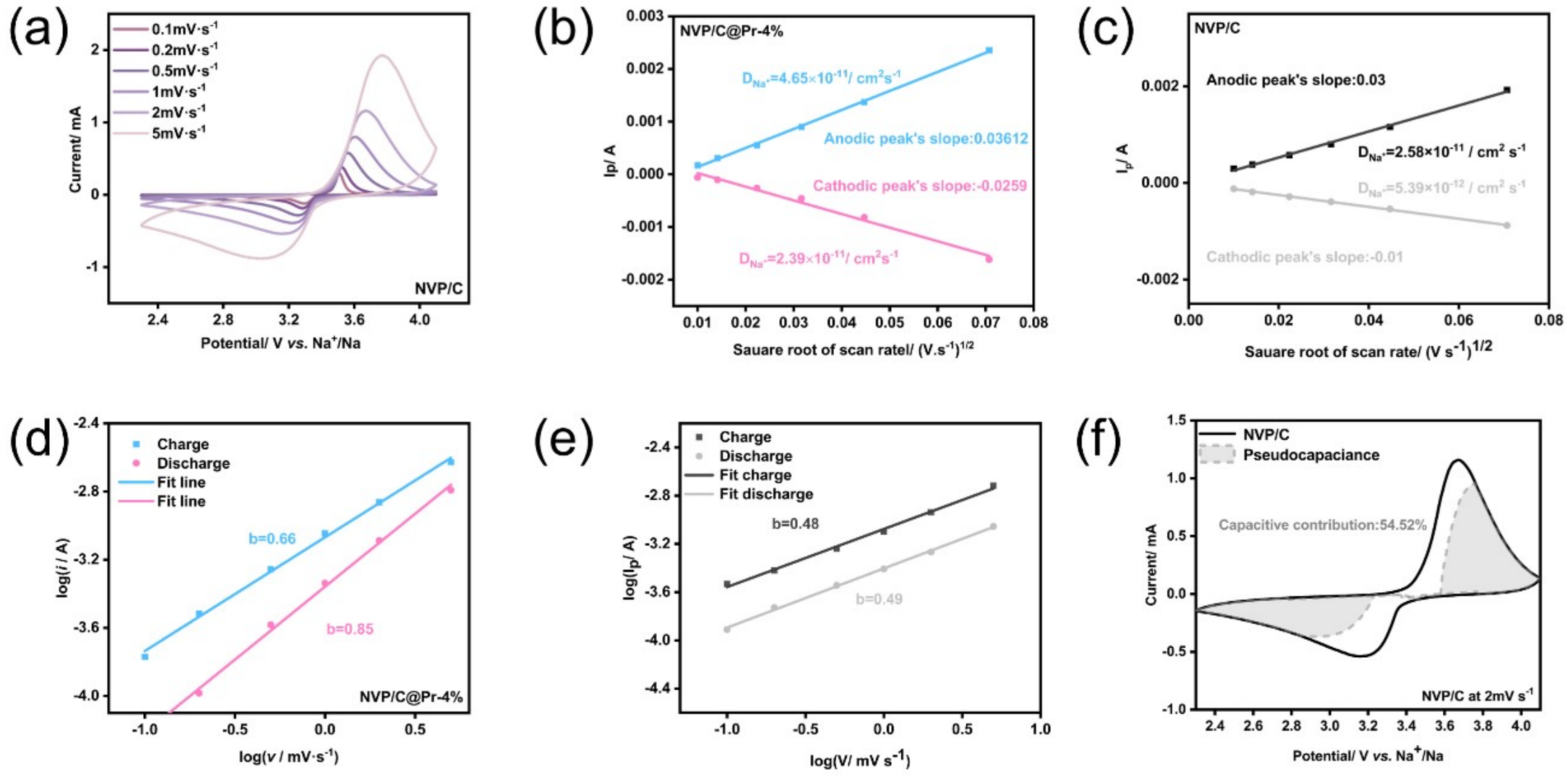

Figure. S4 (a) CV curves of NVP/C at different sweep speeds. (b) D_{Na}^{+} of NVP/C@Pr-4% calculated from CV. (c) D_{Na}^{+} of NVP/C calculated from CV. b-value of (d) NVP/C@Pr-4% and (e) NVP/C (f) Pseudocapacitance contribution of NVP/C samples.

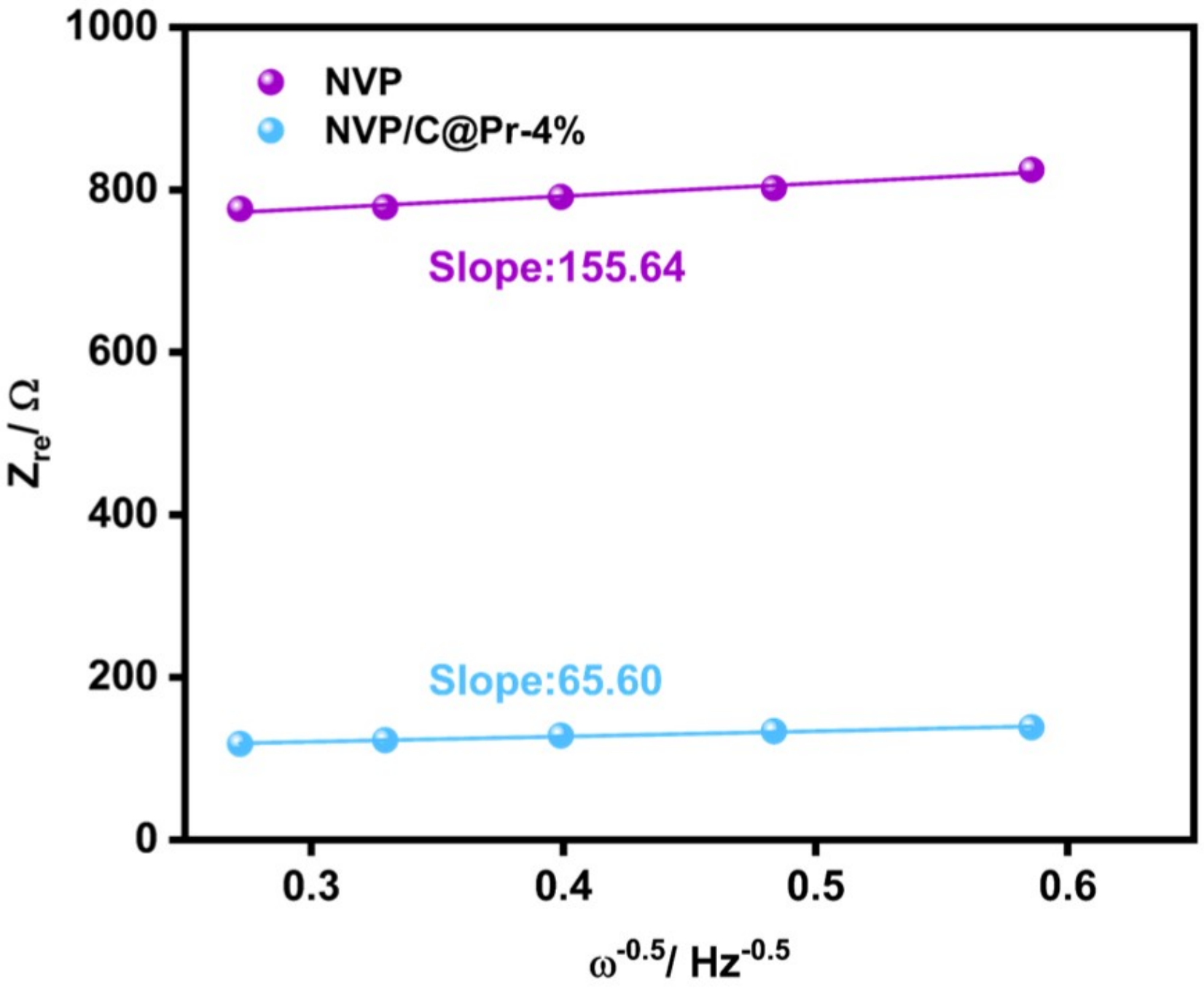

Figure. S5 Comparison of slopes in the low-frequency region of the EIS.

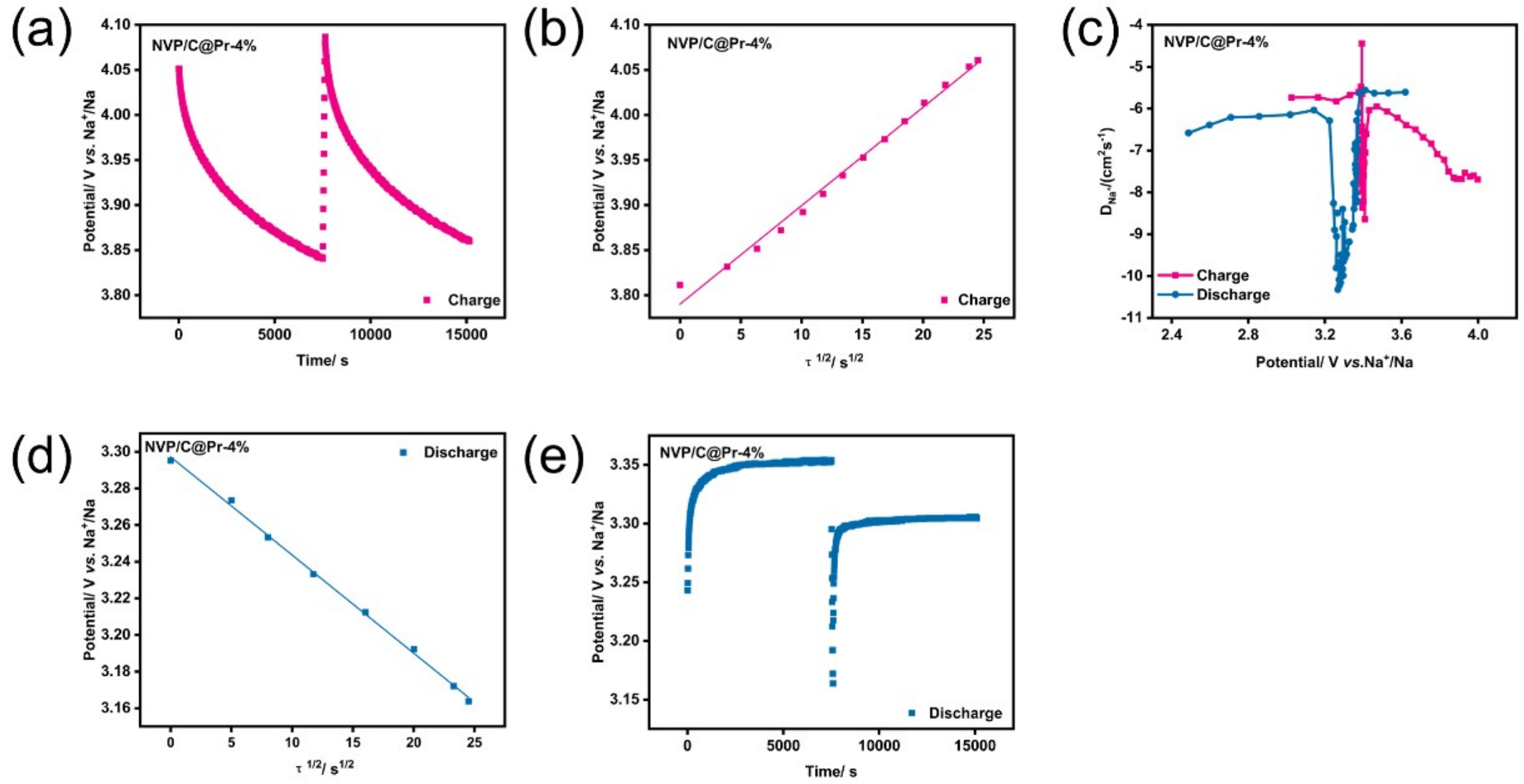

Figure. S6 (a, e) The plot in a single period during charge and discharge process. (b, d) The linear relationship of "v vs. $\tau^{1/2}$" in charge and discharge. (e) Na migration results image of NVP/C@Pr-4%.

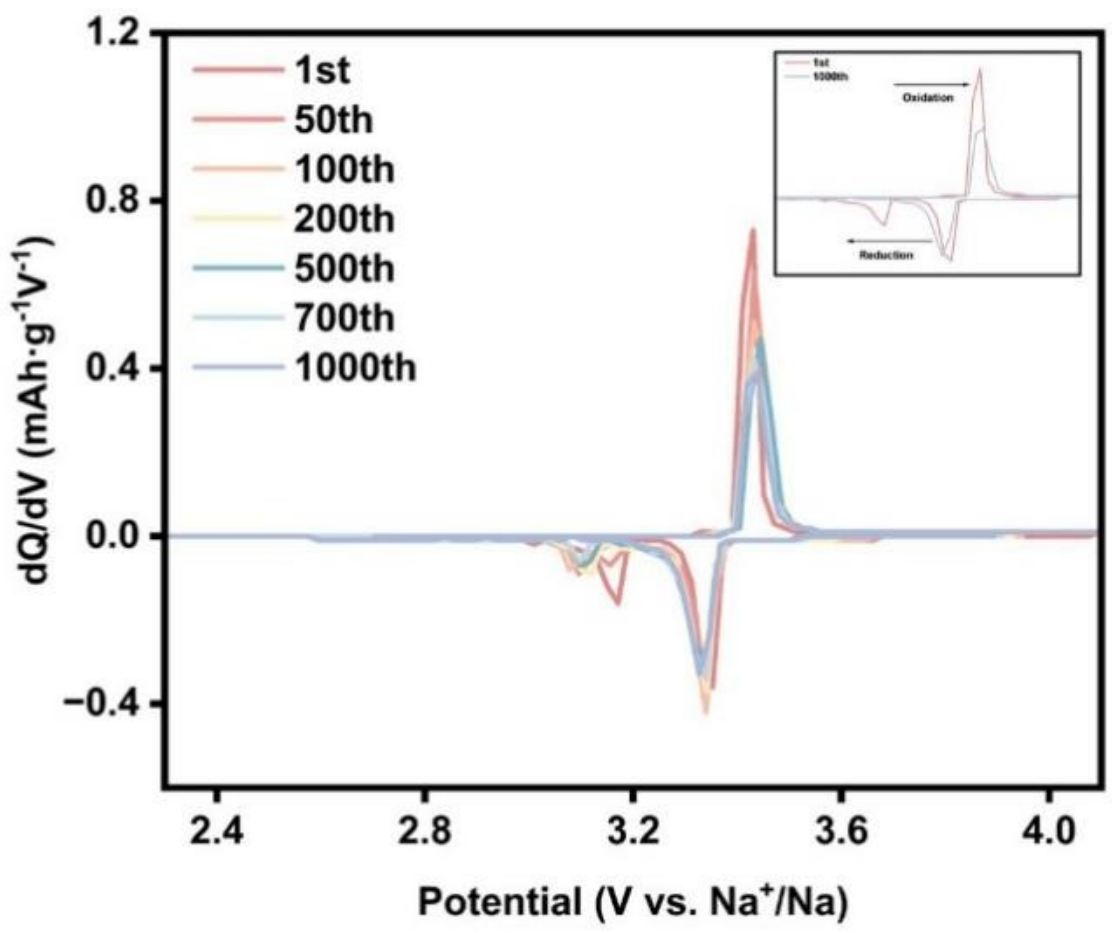

Figure. S7 the corresponding differential capacity (dQ/dV) curves.

Table S1. the results of quantitative EDS mapping for the NVP/C@Pr-4% sample

**Element**	**at%**
Na	3.61
V	6.43
P	10.21
O	41.36
C	36.86
N	1.32
Br	0.05
Pr	0.17

Table S2. The Randles-Sevcik method was used to determine the DNa+ for NVP/C and NVP/C@Pr-4% electrodes

Sample	Slope		D_{Na^+}/ cm^2s^{-1}	
	Charge	Discharge	Charge	Discharge
NVP/C	0.0290	-.0130	$2.97x10^{-11}$	$5.7x10^{-12}$
NVP/C@Pr-4%	0.03612	-0.0259	$4.65x10^{-11}$	$2.39x10^{-11}$

Table S3. The kinetic parameters for NVP/C and NVP/C@Pr-4% electrodes were established through analysis of the EIS measure ments

Sample	R_{ct}/ Ω	σ	D_{Na^+}/ cm^2s^{-1}
NVP/C	750	155.65	1.88x10^{-15}
NVP/C@Pr-4%	95	65.61	1.05x10^{-14}

Table S4. Lattice parameters after fitting by GASA**II.**

**Samples**	**a=b/ Å**	**c/ Å**	**V/ Å³**
**1**	8.73288	21.8042	1438.732
**2**	8.7296	21.80348	1439.056
**3**	8.73363	21.78127	1437.274
**4**	8.73453	21.74557	1433.595
**5**	8.72847	21.71375	1428.665
**6**	8.7278	21.7474	1431.894
**7**	8.72719	21.76232	1436.152
**8**	8.73103	21.77475	1436.681
**9**	8.72983	21.75006	1435.211

Table S5. Comparison of cyling performance between NVP/C@Pr-4% electrode and

advanced phosphates cathodes

Electrode	Intial capacity ($mAhg^{-1}$)/Rate(C)	Cycle Number/Rate (C)	Final capacity ($mAhg^{-1}$)/Rate(C)	References
NVP/C@Pr-4%	105.4/ (1C)	1000/ (1C)	85.1/ (1C)	This work
	85.2/ (10C)	1000/(10C)	83.3/ (10C)	
	81.3/ (30C)	3000/ (30C)	76.7/ (30C)	
	73.8/ (50C)	3000/ (50C)	63.5/ (50C)	
NVP-M⊥	103.2/ (1C)	200/ (1C)	97.5/(1C)	[38]
Cu0.05NVP@C	107.4/(1C)	300/(1C)	102.9/(1C)	[36]
NVP/C-Mo0.04	105.9/ (1C)	200/ (1C)	101/ (1C)	[42]
3DP NVP	110.5/ (1C)	400/ (1C)	102.5/ (1C)	[37]
NV1.8M0.2P	90.43/ (1C)	200/ (1C)	82.98/ (1C)	[40]
NVMTP-1.4	124/ (1C)	200/ (1C)	110.5/(1C)	[39]
NVCP-2	95.4/ (1C)	500/ (1C)	83.1/ (1C)	[34]
NVP/LGy-1.3	101.3/ (0.5C)	400/ (0.5C)	90.4/ (0.5C)	[41]
NVP-YG	115/ (1C)	1000/ (1C)	96.72/ (1C)	[33]
NVFP	155/ (1C)	100/ (1C)	137/ (1C)	[35]

NVP-NFPP	123.6/ (1C)	100/ (1C)	120.02/ (1C)	[43]
NVPF-3NVP/C	112.9/ (1C)	100/ (1C)	96.86/ (1C)	[44]
